# Autophagy hub-protein p62 orchestrates oxidative, endoplasmic reticulum stress, and inflammatory responses post-ischemia, exacerbating stroke outcome

**DOI:** 10.1016/j.redox.2025.103700

**Published:** 2025-05-27

**Authors:** Xingyun Quan, Yukun Yang, Xiaolong Liu, Britta Kaltwasser, Matthias Pillath-Eilers, Bernd Walkenfort, Sylvia Voortmann, Ayan Mohamud Yusuf, Nina Hagemann, Chen Wang, Mike Hasenberg, Dirk M. Hermann, Ulf Brockmeier

**Affiliations:** a Department of Neurology, University Hospital Essen, University of Duisburg-Essen, Germany; b Imaging Center Essen, Institute of Experimental Immunology and Imaging, University Hospital Essen, University Duisburg-Essen, Germany

**Keywords:** Middle cerebral artery occlusion, Oxygen-glucose deprivation, Reoxygenation, Reactive oxygen species, Stress signaling, Apoptosis, Necroptosis, Nuclear factor erythroid-2-related factor-2, Nuclear factor-kB, Protein kinase RNA-Like endoplasmic reticulum kinase

## Abstract

Autophagy has crucial roles for ischemia/reperfusion (I/R) injury. To define the role of the autophagy hub protein p62/SQSTM1 in I/R injury, we conducted gain-of-function and loss-of-function experiments in a set of cell types, including two neuron-like cell lines, primary neurons, brain endothelial and astroglial-like cells, which we combined with mouse ischemic stroke studies. p62 levels post-I/R increased alongside intracellular ROS changes. p62 overexpression increased and p62 knockdown or pharmacological deactivation reduced I/R injury. Autophagic flux was p62-dependent, but oxygen-independent. Using p62 domain deletion mutants we identified p62's ZZ domain as key factor mediating autophagy and cell death. Death-promoting effects of p62 involved elevated ROS burden. At the same time, p62 activated a broad network of cytoprotective responses, which included NRF2-associated antioxidant signaling and inhibition of the pro-inflammatory NFκB pathway, which were bidirectionally linked with p62, and downregulation of the ER stress sensor BiP/GRP78 with consecutive activation of the UPR PERK branch. Our study establishes p62 as a master regulator of I/R injury, which offers itself as target for stroke therapies.

## 1 Introduction

Post-ischemia, tissue reoxygenation (Reox) induces reactive oxygen species (ROS) formation that results in protein misfolding and aggregate formation and exacerbates cell injury [[1], [2], [3]]. The maintenance of neuronal proteostasis is paramount in metabolic stress. While misfolded proteins are cleared by the ubiquitin-proteasome system (UPS), toxic aggregate-prone proteins are mainly eliminated by macroautophagy (hereafter referred to as autophagy) [4]. Therefore, autophagy became a potential therapeutic target in cerebral ischemia/reperfusion (I/R) injury. Cerebral ischemia is a well-known trigger of autophagy [5,6]. It is still debated whether autophagy activation is beneficial or harmful for neurons, as both *in vitro* and *in vivo* studies provided opposing findings regarding the consequences of autophagy for neuronal survival and injury [5,6]. The underlying interactions of autophagy components with other stress pathways are poorly understood. Understanding these interactions is key for an adequate interpretation of the role of autophagy in I/R injury.

The multifunctional adaptor protein p62/SQSTM1 has emerged as a key player in autophagy in a large variety of pathophysiological conditions in recent years [7]. p62 is the central hub protein that targets misfolded ubiquitinated proteins to autophagosomes [7]. For this interaction, p62 possesses its LC3-interacting region (LIR) and ubiquitin-associated (UBA) domains [8,9]. p62 also contains a killer cell immunoglobulin-like receptor (KIR) domain, which mediates its interaction with kelch-like ECH-associated protein-1 (KEAP1), an adaptor protein of the Cul3-ubiquitin E3 ligase complex that targets nuclear factor erythroid-2–related factor-2 (NRF2) for proteasomal degradation [10]. Phosphorylation at Ser351 within the KIRK domain facilitates stronger binding of p62 to KEAP1, thereby stabilizing NRF2 which activates NRF2-dependent antioxidant pathways [10]. Other protein-interaction motifs are the N-terminal Phox and Bem-1 (PB1) domain critical for homo- and hetero-dimerization [11] and the ZZ-type zinc finger domain for interaction with arginylated substrates [12,13]. p62 also contains a tumor necrosis factor receptor-associated factor-6 (TRAF6) binding (TB) domain that regulates K63-linked polyubiquitination of TRAF6, leading to the activation of nuclear factor kappa-light-chain-enhancer of activated B cells (NFκB), a central transcription factor in regulating immune and inflammatory responses [14,15]. A nuclear export signal (NES) and two nuclear localization signals (NLS) convey the nucleo-cytoplasmic shuttling of p62 [16]. Hence, structural components closely define multiple biological functions of this protein.

While recent studies have addressed individual aspects of p62 signaling under I/R conditions [[17], [18], [19], [20]], a detailed analysis of p62's involvement in the complex signaling networks activated by I/R injury is still missing, especially in the context of ischemic stroke. To obtain a comprehensive understanding of p62's role in cellular stress signaling, we chose the two neuron-like cell lines SY5Y and HEK293T and carried out a systematic set of gain-of-function (GOF) and loss-of-function (LOF) experiments using oxygen-glucose deprivation/reoxygenation (OGD/R) as an *in vitro* model of I/R injury. We compared these studies with p62 deactivation experiments in primary neurons, brain endothelial cells and astroglial-like cells and performed transient middle cerebral artery occlusion (MCAO) in mice, which represents a clinically relevant ischemic stroke model *in vivo*. We demonstrate that p62 acts as a driver of I/R injury *in vitro* and *in vivo*, elevating the oxidative burden of cells via autophagy-associated ROS formation and detrimental hypoxia-inducible factor-1α (HIF1α) signaling. Besides, we identified a number of cytoprotective signaling pathways, which included NRF2-associated antioxidant signaling, inhibition of the pro-inflammatory NFκB pathway and downregulation of the endoplasmic reticulum (ER) stress sensor BiP with consecutive activation of the unfolded protein response (UPR) protein kinase RNA-like ER kinase (PERK) pathway. Our studies establish p62 as a potential target for ischemic stroke therapies.

## 2 Results

### 2.1 Post-ischemic reoxygenation triggers p62 accumulation, which modulates apoptotic and necrotic cell death

**Fig. 1:**
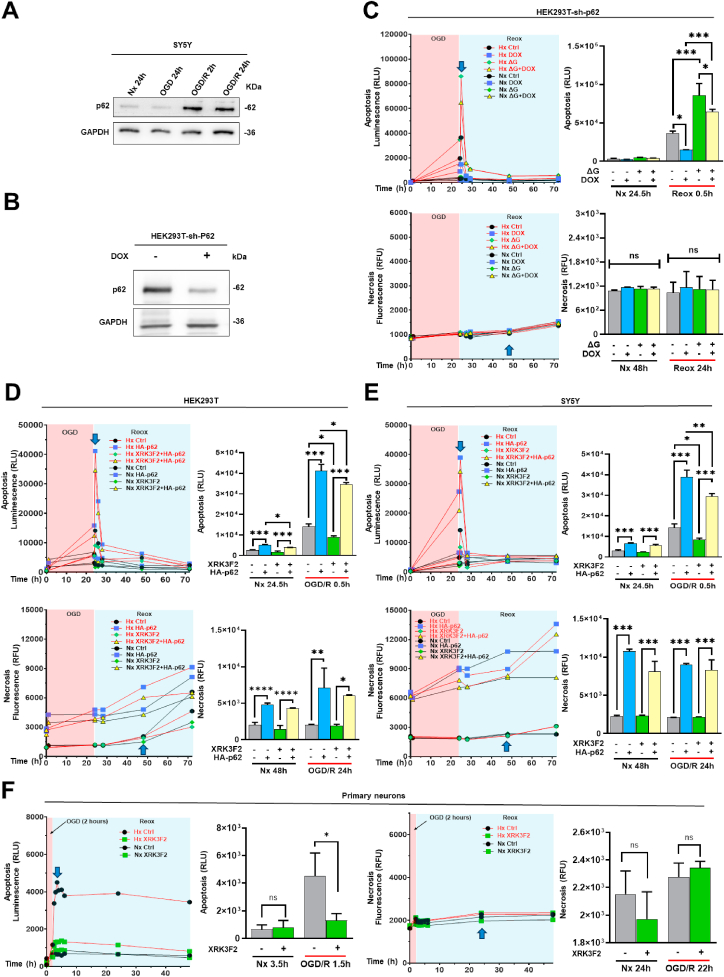
**Loss of p62 function reduces and p62 overexpression increases apoptosis post-ischemia/reoxygenation (I/R). (A)** Western blot analysis of endogenous p62 protein in SY5Y cells exposed to 24 h normoxia (Nx), 24 h oxygen-glucose deprivation (OGD; 1 % O_2_), or 24 h OGD followed by 2 or 24 h reoxygenation/glucose resupplementation (in the following referred to as Reox or OGD/R). **(B)** After inducing p62 knockdown (KD) with doxycycline for 72 h, Western blot analysis was performed in HEK293T cells to assess the p62 KD efficiency. **(C)** Apoptosis and necrosis levels of live HEC293T cells measured by the RealTime-Glo Annexin V Apoptosis and Necrosis Assay. After inducing p62 KD with doxycycline, cells were placed in a Nx (21 % O_2_) or hypoxia (Hx; 1 % O_2_) environment in regular glucose or glucose deprivation (ΔG) conditions for 24 h. Bar graphs display the levels of apoptosis after 30 min Reox and of necrosis after 24 h Reox. **(D, E)** Apoptosis and necrosis levels of live HEK293T and SY5Y cells overexpressing HA-p62 plasmid, which were treated with the p62-ZZ domain inhibitor XRK3F2 at 5 μM during Nx and OGD/R. p62 KD was induced 48 h before the study. HA-p62 plasmid overexpression was induced 24 h before the study. **(F)** Apoptosis and necrosis levels of live primary mouse hippocampal neurons measured by the RealTime-Glo Annexin V Apoptosis and Necrosis Assay. Cells were treated with the p62-ZZ domain inhibitor XRK3F2 at 5 μM and placed in a Nx (21 % O_2_) or Hx (1 % O_2_) environment in regular glucose or glucose deprivation (ΔG) conditions for 24 h. Bar graphs display the levels of apoptosis after 90 min Reox and of necrosis after 24 h Reox. Equal amounts of DMSO (2 %) and pcDNA3.1 plasmid were used as control groups for XRK3F2 and HA-p62 plasmid, respectively. Each experiment was performed three times, with at least three samples processed the same way in each experiment. Data are mean + SD values. Statistical comparisons were performed using two-way ANOVA followed by Tukey's post hoc tests. Statistical significance was indicated as ∗p < 0.05, ∗∗p < 0.01, ∗∗∗p < 0.001, ∗∗∗∗p < 0.0001.

**Fig. 2:**
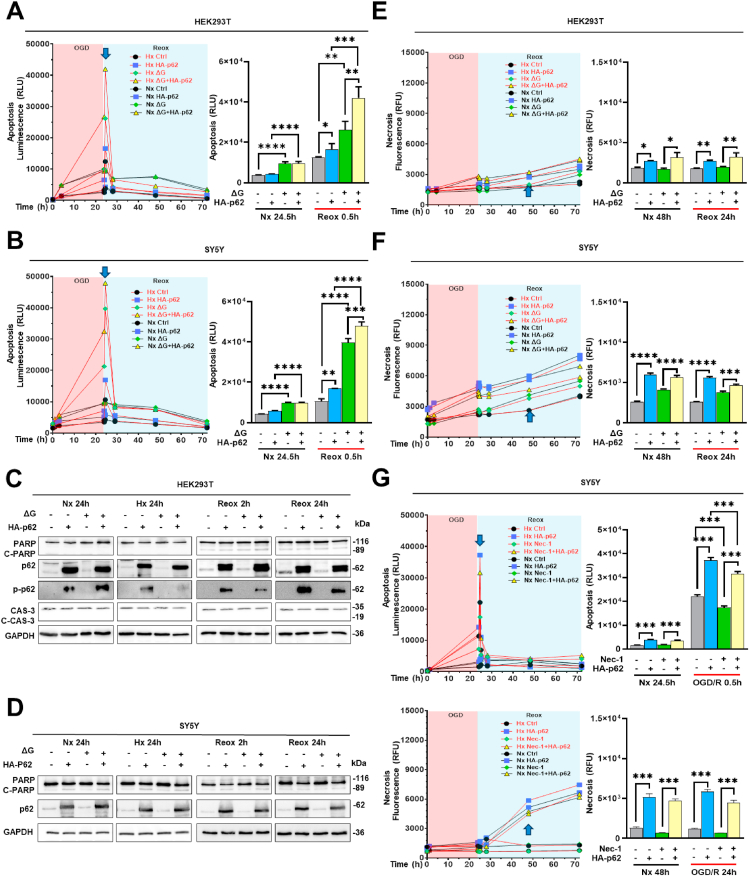
**p62-induced cell death post-I/R is caspase-3 and poly(ADP-ribose)-polymerase (PARP) independent, but partly dependent on the necroptosis inhibitor receptor-interacting protein kinase-1 (RIPK1). (A, B)** Real-time apoptosis levels of HEK293T and SY5Y cells overexpressing HA-p62 plasmid before and after 24 h exposure to a Nx (21 % O_2_) or Hx (1 % O_2_) environment in regular medium or glucose deprivation medium (ΔG). **(C, D)** After oxygen and glucose deprivation followed by 2 or 24 h Reox, caspase-3 (CAS-3) and PARP cleavage were evaluated in HEK293T and SY5Y cells by Western blot analysis, alongside p62 and phospho-p62 (p-p62) abundance. **(E, F)** Necrosis levels of live HEK293T and SY5Y cells overexpressing HA-p62 plasmid before and after 24 h oxygen and glucose deprivation. **(G)** Apoptosis and necrosis levels of live SY5Y cells following treatment with the RIPK1 blocker necrostatin-1 (5 μM), an inhibitor of necroptosis. Equal amounts of DMSO (2 %) were used as control groups for necrostatin-1. HA-p62 plasmid overexpression was initiated 24 h before the study. Plasmid control groups received equal amounts of pcDNA3.1 plasmid. Each experiment was performed three times, with at least three samples simultaneously processed in each experiment. Data are mean + SD values. Statistical comparisons were performed using two-way ANOVA followed by Tukey's post hoc tests. Statistical significance was indicated as ∗p < 0.05, ∗∗p < 0.01, ∗∗∗p < 0.001, ∗∗∗∗p < 0.0001.

We first examined p62 protein responses to I/R in SY5Y cells by Western blots and showed that, compared to normoxia (Nx), p62 levels remained unchanged after 24 h of hypoxia (Hx, 1 % O_2_) and OGD, but increased following Reox, indicating the pathophysiological relevance of p62 in this model system (Fig. 1A). Based on a PiggyBac transposon system, we generated the stable doxycycline-inducible p62 knockdown (KD) cell line HEK293T-sh-p62 and confirmed a KD efficiency of ∼80 % via Western blot (Fig. 1B). We then performed p62 LOF experiments and recorded live imaging of cell death events using the RealTime-Glo® Annexin V Apoptosis and Necrosis assay (Fig. 1C). Following 24 h of OGD we detected an apoptotic peak during early Reox (0.5 h), which was highest after OGD and was significantly reduced by p62 KD. In contrast, cellular necrosis showed a minimal, continuous increase independent from OGD/R or p62 KD. Since we failed to generate the same inducible p62 KD system in SY5Y cells, we repeated the cell death experiments in HEK293T and SY5Y cells exposed to the pharmacological p62 inhibitor XRK3F2 and for the latter studies also included experiments in mouse primary neurons (Fig. 1D–F). In addition, we tested human cerebral microvascular endothelial cells (hCMEC/D3) and human astroglioma cells (U-87 MG) to model key cellular components of the blood–brain barrier, namely endothelial and astroglial cells (Fig. S1). Again, we demonstrated anti-apoptotic effects of pharmacological p62 inhibition following Reox in all tested cell lines which were in line with our prior LOF experiments. Notably, the primary neurons exhibited a continuously elevated apoptotic signal throughout the entire measurement during Reox, which was different from the short-lasting Reox-induced apoptotic peak in immortalized cells. Next, we evaluated the cellular response to exogenous p62 protein expression. The transient transfection of HEK293T and SY5Y cells with plasmid HA-p62, encoding for p62 wildtype fused to a HA-tag at the C-terminus, increased Reox-related apoptosis in both cell lines with the highest peak after OGD (Fig. 2A and B). Western blot analysis revealed increased protein levels not only of total p62, but also of phosphorylated, activated p62 (p-p62) after overexpression (Fig. 2C and D). Interestingly, apoptosis in the early Reox phase was caspase-3-independent, since we did not detect significant caspase-3 or poly ADP-ribose polymerase (PARP) cleavage bands. Further, we noticed that p62 overexpression also induced more cell necrosis, but rather in the late Reox phase (24 h) (Fig. 2E and F). Next, we treated SY5Y cells with the necroptosis inhibitor necrostatin-1 and found a partial reduction in apoptotic signaling (Fig. 2G).

### 2.2 p62 inhibits cell growth in an oxygen-dependent manner

**Fig. 3:**
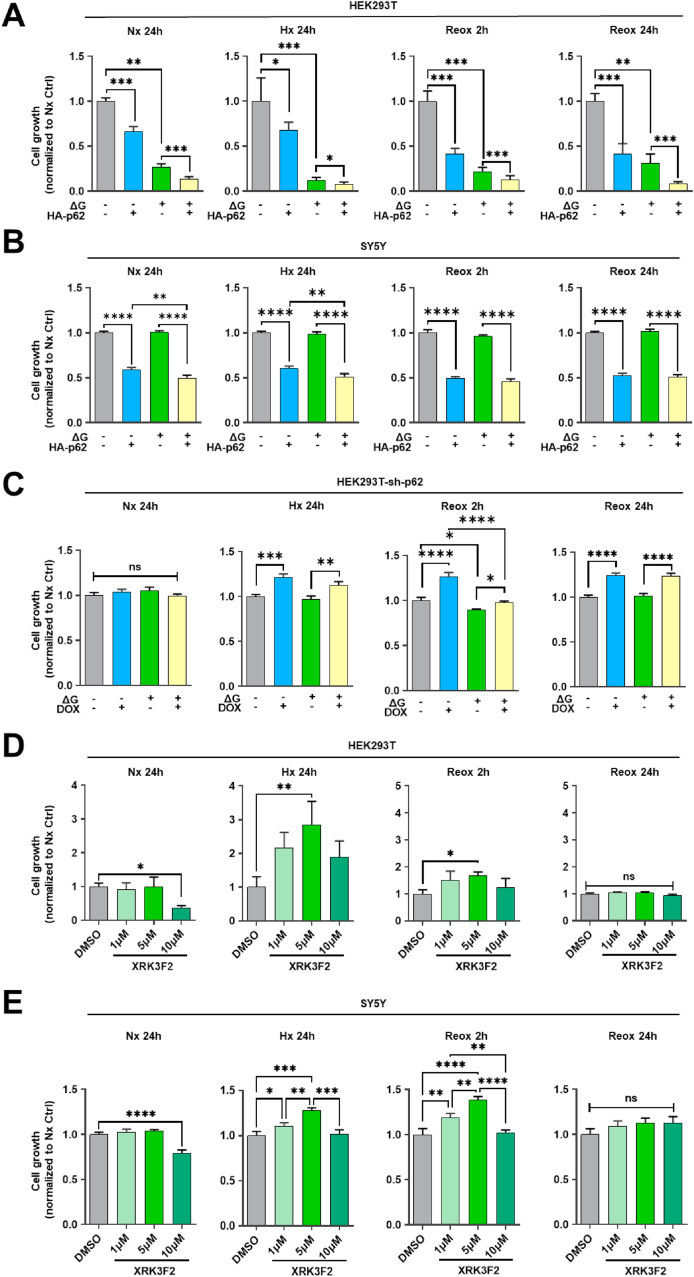
**Effects of p62 modulation on cell proliferation under regular conditions and post-I/R. (A, B)** Proliferation of HEK293T and SY5Y cells exhibiting HA-p62 plasmid overexpression assessed by Coomassie brilliant blue under conditions of normoxia (Nx; 21 % O_2_) or hypoxia (Hx; 1 % O_2_) in regular medium or glucose deprivation medium (ΔG), followed by different durations of Reox. The bar graphs represent normalized cell growth. **(C)** Proliferation of HEK293T cells exhibiting shRNA-mediated p62 KD under various conditions specified above. p62 KD was induced by doxycycline 72 h before the study. **(D, E)** Proliferation of HEK293T and SY5Y cells after exposure to different XRK3F2 concentrations under various conditions specified above. Equal amounts of DMSO (2 %) were used as control groups for XRK3F2. HA-p62 plasmid overexpression was initiated 24 h before the study. Plasmid control groups received equal amounts of pcDNA3.1 plasmid. Each experiment was performed three times, with at least three samples simultaneously processed in each experiment. Data are mean + SD values. Statistical comparisons were performed using two-way ANOVA followed by Tukey's post hoc tests. Statistical significance was indicated as ∗p < 0.05, ∗∗p < 0.01, ∗∗∗p < 0.001, ∗∗∗∗p < 0.0001.

OGD is known to impair cellular energy metabolism, increase autophagic cell death and activate oxidative stress [21]. Hence, we tested the effect of p62 overexpression on the proliferation of HEK293T and SY5Y cells exposed to OGD (Fig. 3A and B). Regardless of oxygen or glucose deprivation, we found a strong inhibition of cell growth by p62 overexpression, whereas p62 KD increased the growth of HEK293T cells experiencing Hx (Fig. 3C). At the same time point, p62 KD did not influence Nx cell growth. Based on a previous study investigating XRK3F2 inhibition in cell culture [22], we examined the effects of various concentrations of the pharmacological p62 inhibitor XRK3F2 on cell growth. Our data show that XRK3F2 at a concentration of 5 μM promotes the proliferation of Hx and early Reox (2 h), but not of Nx or late Reox (24 h) HEK293T and SY5Y cells. Of note, a higher concentration of 10 μM XRK3F2 was toxic and impaired Nx cell growth (Fig. 3D and E). The Coomassie brilliant blue staining figures are compiled in the supplementary file (Fig. S1 A-E).

### 2.3 p62 controls autophagic flux post-I/R

**Fig. 4:**
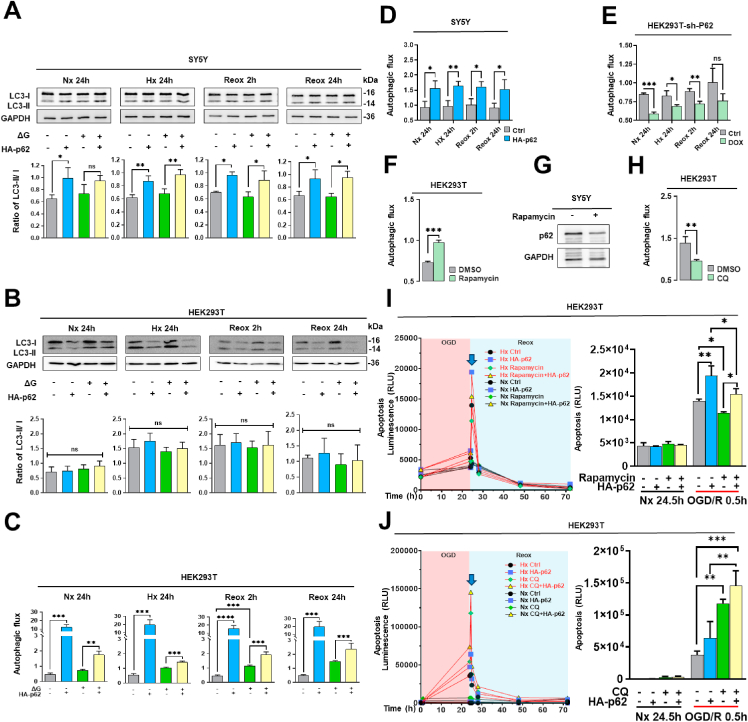
**Effects of p62 on autophagy proteins and autophagic flux under regular conditions and post-I/R. (A, B)** Western blot analysis of LC3 protein in SY5Y and HEK293T cells overexpressing HA-p62 plasmid, which were exposed to various oxygen and glucose deprivation conditions. **(C)** Autophagic flux assessed using a luciferase-based autophagic flux reporter assay in HEK293T cells overexpressing HA-p62 plasmid under various oxygen and glucose deprivation conditions. **(D, E)** Autophagic flux measured using the luciferase-based autophagic flux reporter assay in HEK293T and SY5Y cells exhibiting siRNA-induced p62 KD which were exposed to different Hx and Reox conditions. **(F)** Following treatment of HEK293T cells with the autophagy inducer rapamycin (1 μM) for 24 h, autophagic flux was again assessed using the luciferase-based autophagic flux reporter assay. **(G)** p62 protein level examined by Western blot analysis in SY5Y cells treated with the autophagy inducer rapamycin at a concentration of 1 μM (2 % DMSO served as control group). **(H)** After incubating cells with the late-stage autophagy inhibitor chloroquine (CQ; 10 μM) for 24 h, autophagic flux was evaluated using the fluorescence-based luciferase reporter assay. **(I, J)** Apoptosis levels of HEK293T cells overexpressing HA-p62 plasmid which were exposed to Nx or OGD/R and treated with rapamycin or chloroquine (CQ). HA-p62 plasmid overexpression was initiated 24 h before the study. Plasmid control groups received equal amounts of pcDNA3.1. Each experiment was performed three times, with at least three samples simultaneously processed in each experiment. Data are mean + SD values. Statistical comparisons between two groups were performed using Student's t-tests, comparisons between four or more groups were performed using two-way ANOVA followed by Tukey's post hoc tests. Statistical significance was indicated as ∗p < 0.05, ∗∗p < 0.01, ∗∗∗p < 0.001, ∗∗∗∗p < 0.0001.

The excessive expression of p62 is closely associated with autophagy dysregulation in various disease conditions [23]. Hence, we investigated the effect of p62 GOF and LOF on autophagy under I/R conditions. To assess autophagy, we first analyzed the lipidation of microtubule-associated protein-1A/1B-light chain-3 (LC3) by Western blot (Fig. 4A and B). p62 overexpression increased LC3-II/I ratio independent of the oxygen level in SY5Y cells, indicating autophagy activation (Fig. 4A). In HEK293T cells, however, LC3 lipidation was increased by oxygen deprivation but hardly by exogenous p62 expression (Fig. 4B). Due to this inconsistency between both cell lines and the fact that induction of autophagy does not necessarily reflect the autophagic flux, we decided to use a different approach. We conducted a luciferase-based assay that enabled us to detect the lysosomal degradation of lipidated LC3-II and found that in both cell lines p62 strongly augmented autophagic flux independent of the oxygen level (Fig. 4C and D). We could also confirm that additional glucose deprivation further increased autophagic flux (Fig. 4C). On the other hand, p62 KD in HEK293T cells severely reduced autophagic flux in I/R conditions independent of oxygen (Fig. 4E). To investigate the potential link between autophagy and cell apoptosis, we used rapamycin, a potent activator of autophagy by inhibiting the mechanistic target of rapamycin (mTOR), and chloroquine (CQ), a compound that inhibits lysosomal function by blocking the fusion of autophagosomes with lysosomes. In SY5Y cells, rapamycin reduced the levels of endogenous p62 protein (Fig. 4G). In HEK293T cells, rapamycin increased autophagic flux, whereas CQ decreased autophagic flux (Fig. 4F–H). The Reox-induced apoptotic peak was significantly decreased by rapamycin independent of exogenous p62 expression (Fig. 4I). In line with this result, we observed a dramatic increase of apoptosis after CQ incubation (Fig. 4J). In these studies, CQ treatment alone more strongly increased apoptosis than p62 overexpression alone.

### 2.4 Role of p62 domains in autophagic flux modulation and cell fate post-I/R

**Fig. 5:**
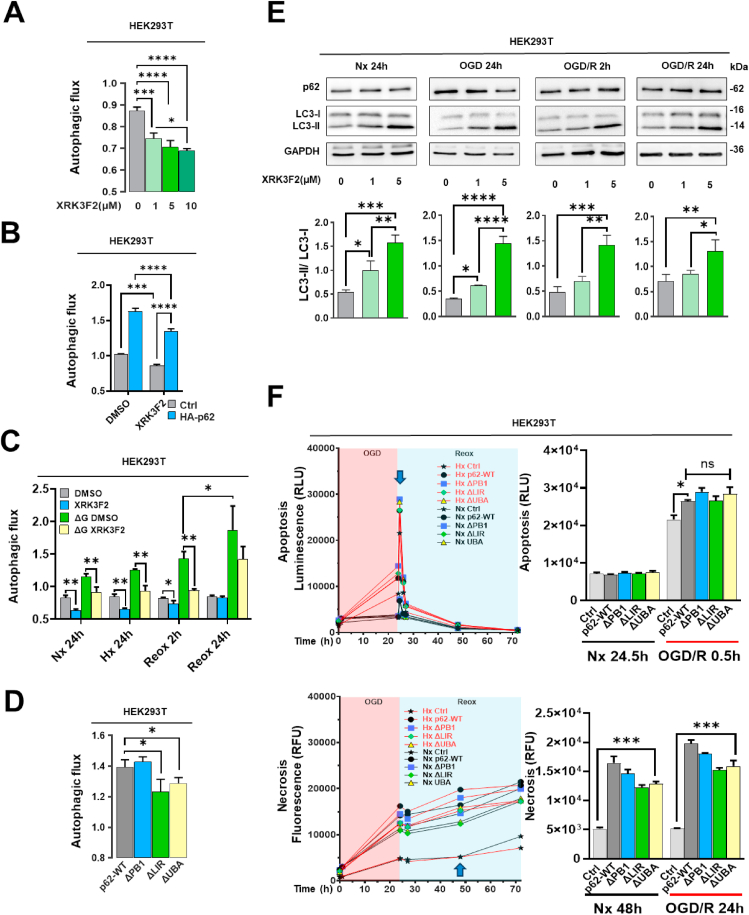
**Domain-specific effects of p62 on cell apoptosis, necrosis, and autophagic flux post-I/R. (A)** Autophagic flux measured by the fluorescence-based luciferase reporter assay in HEK293T cells treated with different concentrations of the p62-ZZ domain inhibitor XRK3F2. **(B, C)** Autophagic flux assessed by the fluorescence-based luciferase reporter assay in HA-p62 plasmid overexpressing HEK293T cells treated with XRK3F2 (5 μM) and HEK293T cells treated with XRK3F2 (5 μM) after exposure to various oxygen and glucose deprivation conditions. HA-p62 plasmid overexpression was initiated 24 h before the study. **(D)** To investigate the functional roles of various structural domains of the p62 protein, we overexpressed p62 proteins with individually deleted PB1, LIR, and UBA domains in HEK293T cells. After 24 h of overexpression of truncated p62 proteins, autophagic flux was assessed by the fluorescence-based luciferase reporter assay. **(E)** Western blot analysis of p62 and LC3 proteins in HEK293T cells treated with different concentrations of XRK3F2, which were exposed to Nx, OGD or OGD/R. Bar graphs show the ratio of LC3II to LC3I protein. **(F)** Real-time apoptosis and necrosis levels of HEK293T cells overexpressing p62 proteins with deleted PB1, LIR, and UBA domains, which were exposed to Nx or OGD/R. Plasmid control groups received equal amounts of pcDNA3.1. Each experiment was performed three times, with at least three samples simultaneously processed in each experiment. Data are mean + SD values. Statistical comparisons were performed using one-way or two-way ANOVA, as adequate, followed by Tukey's post hoc tests. Statistical significance was indicated as ∗p < 0.05, ∗∗p < 0.01, ∗∗∗p < 0.001, ∗∗∗∗p < 0.0001.

p62 is a multifunctional protein, whose functions are determined by its various domains. The ZZ domain was suggested to facilitate autophagy by p62 binding to LC3 [24]. Indeed**,** our autophagic flux assay demonstrated that the specific ZZ domain inhibitor XRK3F2 suppressed autophagic flux at concentrations ranging from 1 to 10 μM in HEK293T cells (Fig. 5A). A XRK3F2 concentration of 5 μM also partially inhibited the increased autophagic flux induced by exogenous p62 (Fig. 5B). XRK3F2 also reduced autophagic flux induced by glucose deprivation during early Reox (Fig. 5C). Interestingly, Western blot analysis showed that XRK3F2 increased the LC3-II/I ratio under Nx, OGD and OGD/R conditions, indicating that LC3-II accumulation can be a sign of autophagic flux inhibition (Fig. 5E). To investigate the functional role of various p62 domains in I/R-related cell death, PB1, LIR and UBA domain-deleted constructs were overexpressed and compared to full length p62 protein in HEK293T cell lines. Autophagic flux assays revealed that in contrast to the PB1 mutant, the absence of LIR and UBA domains reduced the autophagic process (Fig. 5D). Under I/R conditions, however, overexpression of all p62 deletion mutants lacking the LIR, UBA and PB1 domains increased early Reox apoptosis and late Reox necrosis (Fig. 5F). The results indicate that these three domains are not the primary factors causing p62-induced cell death. However, we observed that XRK3F2 reduced Reox-related apoptosis in both SY5Y and HEK293T cell lines (Fig. 1D and E). This finding strongly indicates that the ZZ domain is the critical factor not only for autophagy but also for apoptotic cell death.

### 2.5 p62 increases mitochondrial reactive oxygen species (ROS) production post-I/R in a NOX-independent manner and triggers autophagic cell death

**Fig. 6:**
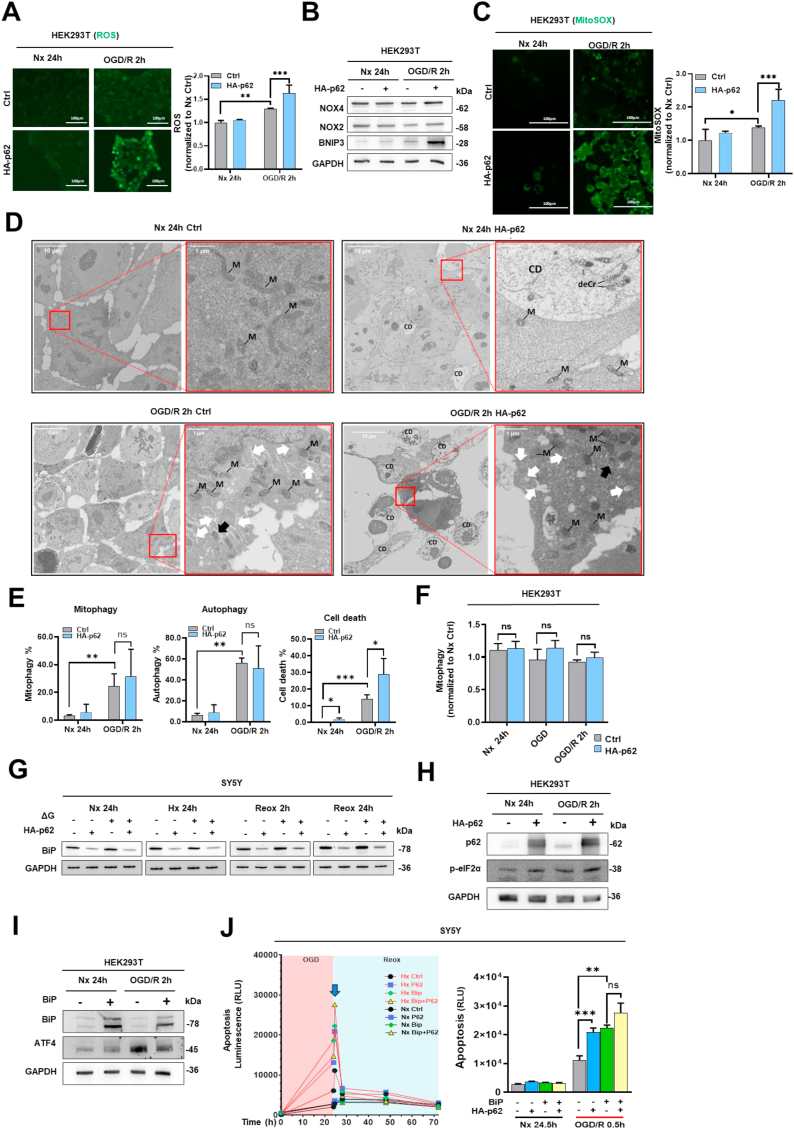
**Effects of p62 and BiP on oxidative stress, autophagy/mitophagy and cell death post-I/R. (A)** Cellular ROS production evaluated by the fluorescent dye CellROX Green in HEK293T cells overexpressing HA-p62 plasmid, which were exposed to 24 h Nx or 24 h OGD followed by 2 h Reox (OGD/R). CellROX is non-fluorescent under normal conditions but generates green fluorescence when oxidized, which was subsequently quantified. **(B)** Western blot analysis of NOX2, NOX4 and BNIP3 proteins in HA-p62 plasmid overexpressing HEK293T cells exposed to 24 h Nx or 24 h OGD followed by 2 h Reox. **(C)** Mitochondrial ROS production assessed by the fluorescent dye MitoSOX in HA-p62 plasmid overexpressing HEK293T cells, which were exposed to 24 h Nx or 24 h OGD followed by 2 h Reox. MitoSOX is non-fluorescent under normal conditions but generates green fluorescence when oxidized by mitochondrial superoxide, which was quantified. HA-p62 overexpression was initiated 24 h before the study. pcDNA3.1 plasmid was used as control group for HA-p62 plasmid. **(D)** Transmission electron microscopy (TEM) images of control and HA-p62 plasmid overexpressing HEK293T cells exposed to 24 h Nx or 24 h OGD followed by 2 h Reox (OGD/R). Highlighted are mitochondria (M), deformed cristae (deCr), dying/dead cells (CD), autophagosomal structures (white arrow) and autophagosomal structures with fragmented mitochondria (black arrow), n = 3 independent experiments. Representative images are shown for each condition. **(E)** TEM images from (D) were analyzed for autophagy, mitophagy and cell death. Autophagic cells were identified by the presence of autophagosomes, double-membraned, electron-lucent vesicles. Mitophagic cells were identified by mitochondria partially or fully enclosed by autophagosomes. Dying/dead cells were identified by their presence of disrupted cellular architecture. A total of ≥220 cells were assessed for each condition. **(F)** Mitophagy assessed by flow cytometry in control and HA-p62 plasmid overexpressing HEK293T cells co-transfected with mKeima-Red-Mito-7, which were exposed to 24 h Nx, 24 h OGD or 24 h OGD followed by 2 h Reox. **(G)** Western blot analysis of BiP protein in control and HA-p62 plasmid overexpressing SY5Y cells exposed to 24 h Nx, OGD or OGD followed by 2 h Reox. **(H)** Western blot analysis of p62 and *p*-eIF2α proteins in control and HA-p62 plasmid overexpressing HEK293T cells exposed to 24 h Nx or 24 h OGD followed by 2 h Reox. **(I)** Western blot analysis of BiP and ATF4 BiP proteins in control and BiP plasmid overexpressing HEK293T cells exposed to 24 h Nx or 24 h OGD followed by 2 h Reox. In (G)–(I), GAPDH was used as loading control. HA-p62 and BiP plasmid overexpression were initiated 24 h before the study. pcDNA3.1 plasmid was used as control group. **(J)** Real-time apoptosis levels of SY5Y cells co-transfected with pcDNA3.1 control, HA-p62 and BiP plasmids following exposure to 24 h normoxia (Nx) or 24 h OGD followed by 2 h Reox (OGD/R). Each experiment was performed three times, with at least three samples simultaneously processed in each experiment. Data are mean + SD values. Statistical comparisons were performed using two-way ANOVA, followed by Tukey's post hoc tests. Statistical significance was indicated as ∗p < 0.05, ∗∗p < 0.01, ∗∗∗p < 0.001.

During Reox, the reentry of oxygen leads to massive ROS production, which can damage proteins and DNA and trigger apoptosis or necrosis [25]**.** To explore the effect of p62 on cellular ROS, we used the intracellular ROS detection reagent CellROX, which emits a stable green fluorescent signal once oxidized and is therefore suitable for visual quantification. In HEK293T cells we found that ROS levels were elevated after 2 h Reox compared to Nx and that p62 further increased ROS levels (Fig. 6A). Further Western blot experiments showed that NADPH oxidase-2 (NOX2) and NADPH oxidase-4 (NOX4), two important enzymes responsible for generation of intracellular ROS, were not upregulated by p62 (Fig. 6B). However, we detected a moderate increase of the pro-apoptotic/autophagic protein BNIP3 [26] after p62 overexpression, while the combination with OGD/R further triggered its accumulation (Fig. 6B). MitoSOX is a fluorescent probe specifically designed to detect levels of mitochondrial ROS. It enters live cells and is selectively oxidized by superoxide radicals within mitochondria, emitting green fluorescence [27]**.** We found that mitochondrial ROS levels were also increased after 2 h Reox and that p62 further increased mitochondrial ROS levels (Fig. 6C). Mitochondrial damage may prompt cells to initiate mitophagy to remove the injured mitochondria. Therefore, we conducted transmission electron microscopy (TEM) to identify fragmented mitochondria associated with autophagosomes (Fig. 6D and E). Compared to Nx, where overexpression of p62 only slightly increased cell death without significant augmentation of autophagy, we detected a dramatic upregulation of autophagosomal structures and dead cells after OGD/R. We also noticed a significant increase in mitophagic cells. However, the recorded mitophagic events/cell were constantly few (≤3) regarding the huge cellular pool of mitochondria. Additional flow cytometry analysis confirmed that p62 did not significantly change the mitophagy level in HEK293T cells (Fig. 6F).

### 2.6 p62 activates endoplasmic reticulum (ER) stress pathway PERK post-I/R

Oxidative stress caused by ROS disrupts protein folding in the ER, inducing ER stress [28]. BiP is an ER-luminal chaperone and UPR stress sensor [29]. Western blot analysis showed that p62 decreased BiP levels in an Hx and Reox independent way (Fig. 6G). To check if the p62-dependent depletion of BiP triggers the UPR PERK branch, we analyzed the phosphorylation status of PERK downstream target eIF2α and could confirm its increase after p62 overexpression (Fig. 6H). Interestingly, BiP overexpression decreased the PERK downstream target ATF4, but only after OGD/R indicating the inhibition of PERK activation under these conditions (Fig. 6I). Since overexpressed BiP also increased Reox-associated apoptosis (Fig. 6J), we conclude that p62-mediated UPR stress activation via PERK functions as a cell-protective mechanism.

### 2.7 p62 modulates cellular NRF2 activity, thus stimulating antioxidant responses

**Fig. 7:**
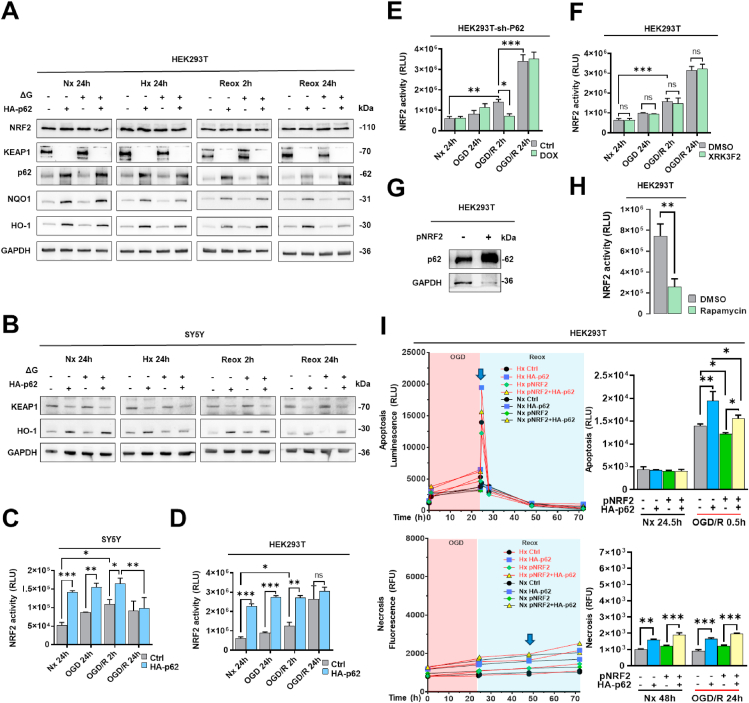
**Roles of the KEAP1-NRF2 pathway in mediating signaling responses of p62 post-I/R. (A, B)** Western blot analysis of NRF2, KEAP1, p62, NQO1, and HO-1 proteins in HEK293T and SY5Y cells overexpressing HA-p62 plasmid, which were exposed to various oxygen and glucose deprivation conditions. GAPDH was used as loading control. **(C, D)** NRF2 activity assessed by the antioxidant response element (ARE)-based firefly luciferase reporter system in HA-p62 plasmid overexpressing SY5Y and HEK293T cells under conditions of Nx, OGD or OGD followed by 2 or 24 h Reox (OGD/R). **(E, F)** Likewise, NRF2 activity was measured using the firefly luciferase reporter system in HEK293T cells exposed to shRNA-induced p62 KD (sh-p62) (in E) or pharmacological p62 deactivation using the ZZ domain inhibitor XRK3F2 (5 μM) (in F) under Nx, OGD or OGD followed by 2 or 24 h Reox conditions. **(G)** Western blot analysis of p62 protein after stable pNRF2 plasmid overexpression in HEK293T cells. GAPDH was used as loading control. **(H)** NRF2 activity evaluated using the firefly luciferase reporter system in HEK293T cells treated with the autophagy inducer rapamycin (1 μM) for 24 h. **(I)** Real-time apoptosis and necrosis level of HEK293T cells overexpressing stable pNRF2 or HA-p62 plasmid under Nx and OGD/R conditions. HA-p62 and pNRF2 plasmid overexpression were initiated 24 h before the study. Plasmid control groups received equal amounts of pcDNA3.1. Each experiment was performed three times, with at least three samples simultaneously processed in each experiment. Statistical comparisons between two groups were performed using Student's t-tests, comparisons between four or more groups were performed using two-way ANOVA followed by Tukey's post hoc tests. Statistical significance was indicated as ∗p < 0.05, ∗∗p < 0.01, ∗∗∗p < 0.001.

Under physiological conditions, the transcription factor NRF2 resides in the cytoplasm, where it forms a complex with kelch-like ECH-associated protein-1 (KEAP1) that prevents the nuclear translocation and activation of NRF2 [30]**.** Under I/R conditions, Western blot analysis showed that p62 overexpression eliminated KEAP1 protein in SY5Y and HEK293T cells and induced the formation of its two major downstream targets NAD(P)H quinone oxidoreductase-1 (NQO1) and heme oxygenase-1 (HO1) (Fig. 7A and B). Luciferase-based gene reporter assays showed that p62 overexpression increased NRF2 activity during Nx, OGD and in the early Reox phase (2 h), whereas p62 KD reduced NRF2 activity in the early Reox phase (2 h) (Fig. 7C–E). However, the p62 inhibitor XRK3F2 had no effect on NRF2 activity (Fig. 7F). Interestingly, NRF2 overexpression increased the levels of endogenous p62 protein (Fig. 7G). In addition, the autophagy activator rapamycin reduced NRF2 activity in Nx (Fig. 7H). Moreover, OGD/R also increased NRF2 activity (Fig. 7C–F), which may represent a protective response of cells under oxidative stress conditions. Overexpression of NRF2 reduced Reox-induced apoptosis and attenuated the pro-apoptotic effect of p62 in HEK293T cells (Fig. 7I).

### 2.8 p62 regulates inflammatory responses via bidirectional negative feedback loop with NFκB

**Fig. 8:**
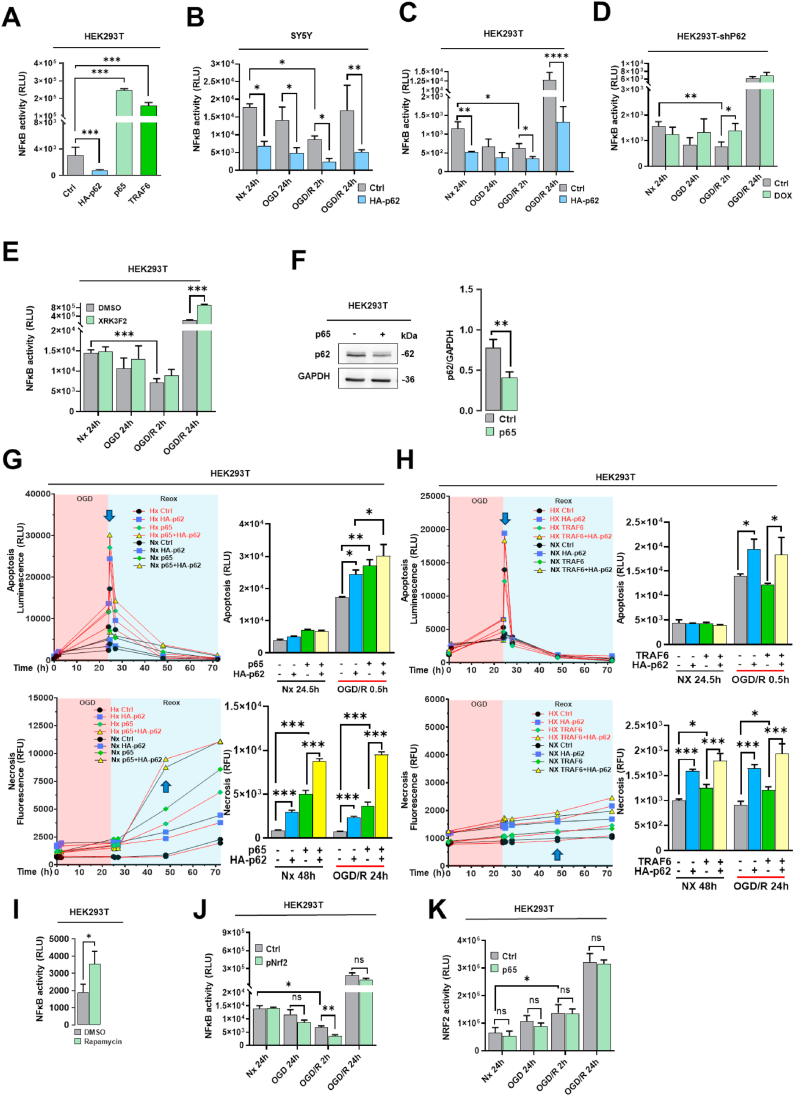
**Roles of NFκB in mediating p62 signaling responses post-I/R. (A)** NFκB activity assessed by a firefly luciferase-based gene reporter assay in HEK293T cells overexpressing HA-p62 plasmid, with p65 and TRAF6 overexpression serving as positive controls. **(B, C)** NFκB activity assessed by the firefly luciferase reporter system in HA-p62 plasmid overexpressing SY5Y and HEK293T cells exposed to Nx, OGD or OGD followed by Reox (OGD/R). **(D)** NFκB activity measured by the firefly luciferase reporter system in HEK293T cells exhibiting shRNA-induced p62 KD, which were exposed to Nx, OGD or OGD/R. **(E)** NFκB activity evaluated by the firefly luciferase reporter system in HEK293T cells treated with the p62-ZZ domain inhibitor XRK3F2 (5 μM), which were exposed to Nx, OGD or OGD/R. **(F)** Western blot analysis of p62 protein after p65 overexpression in HEK293T cells. **(G)** Real-time apoptosis and necrosis levels of HEK293T cells overexpressing TRAF6 and HA-p62 plasmids under Nx and OGD/R conditions. **(H)** Real-time apoptosis and necrosis levels of HEK293T cells overexpressing p65 and HA-p62 plasmids under Nx and OGD/R conditions. **(I)** After incubating HEK293T cells with the autophagy inducer rapamycin (1 μM), NFκB activity was assessed by the firefly luciferase reporter system. (**J**) In HEK293T cells overexpressing stable pNRF2 plasmid, NFκB activity was examined using the firefly luciferase reporter system under Nx, OGD and OGD/R conditions. **(K)** Conversely, in HEK293T cells overexpressing p65 plasmid, NRF2 activity was measured using the firefly luciferase reporter system under Nx, OGD and OGD/R conditions. Plasmid overexpression was initiated 24 h before the study. Plasmid control groups received equal amounts of pcDNA3.1. Each experiment was performed three times, with at least three samples simultaneously processed in each experiment. Statistical comparisons between two groups were done using Student's t-tests, comparisons between four or more groups were performed using one-way or two-way ANOVA followed by Tukey's post hoc tests. Statistical significance was indicated as ∗p < 0.05, ∗∗p < 0.01, ∗∗∗p < 0.001, ∗∗∗∗p < 0.0001.

The transcription factor NFκB controls immune responses, inflammation, and cell survival [31]. Although p62 was shown to regulate NFκB signaling in tumor cells via interaction with receptor-interacting protein-1 (RIP1) and TNF receptor-associated factor-6 (TRAF6), its role in the context of autophagy is still debated [32]. Thus, we evaluated the role of p62 for the inflammatory response post-I/R. Initially, we employed our luciferase-based gene reporter assay for measuring NFκB activity in HEK293T cells and confirmed its functionality by transfection with NFκB subunit p65 or TRAF6 encoding plasmids that triggered a high NFκB response (Fig. 8A). In contrast, exogenous p62 expression strongly reduced NFκB luciferase activity in Nx (Fig. 8A). We next applied the same approach to I/R conditions and noticed that p62 reduced NFκB activity regardless of OGD and Reox in SY5Y and HEK293T cells (Fig. 8B and C). Interestingly, p62 KD increased NFκB activity in OGD and early (2 h) Reox (Fig. 8D). Although chemical inhibition of p62 with XRK3F2 showed the same tendencies, the NFκB activation got only significant in the late (24 h) Reox phase (Fig. 8E). In addition, we found that the negative effect of p62 on NFκB is bidirectional as overexpressed NFκB subunit p65 clearly decreased p62 protein levels (Fig. 8F). p65 GOF also increased Reox-mediated apoptosis and late-stage necrosis in an OGD- and Reox-independent way (Fig. 8G). Of note, p62 might also interact upstream of NFκB with TRAF6 regulating the inflammatory response post-I/R. Yet, compared to our p65 GOF results, we could demonstrate that overexpressed TRAF6 did not trigger early Reox-related apoptosis but boosted cell necrosis in the late (24 h) Reox phase (Fig. 8H). In contrast to the effect of increased p62 levels (Fig. 8A–C), incubation with the autophagy activator rapamycin increased the NFκB activity indicating that autophagic flux after p62 expression is not responsible for the depleted inflammatory response (Fig. 8I). Furthermore, NRF2 had no effect on NFκB activity in Nx, but reduced NFκB activity in early (2 h) Reox (Fig. 8J) and Reox-mediated apoptosis (Fig. 7I). Notably, although overexpression of p65 increased necrosis (Fig. 8G), it had no effect on NRF2 activity (Fig. 8K).

### 2.9 p62 promotes cell death via HIF1α

**Fig. 9:**
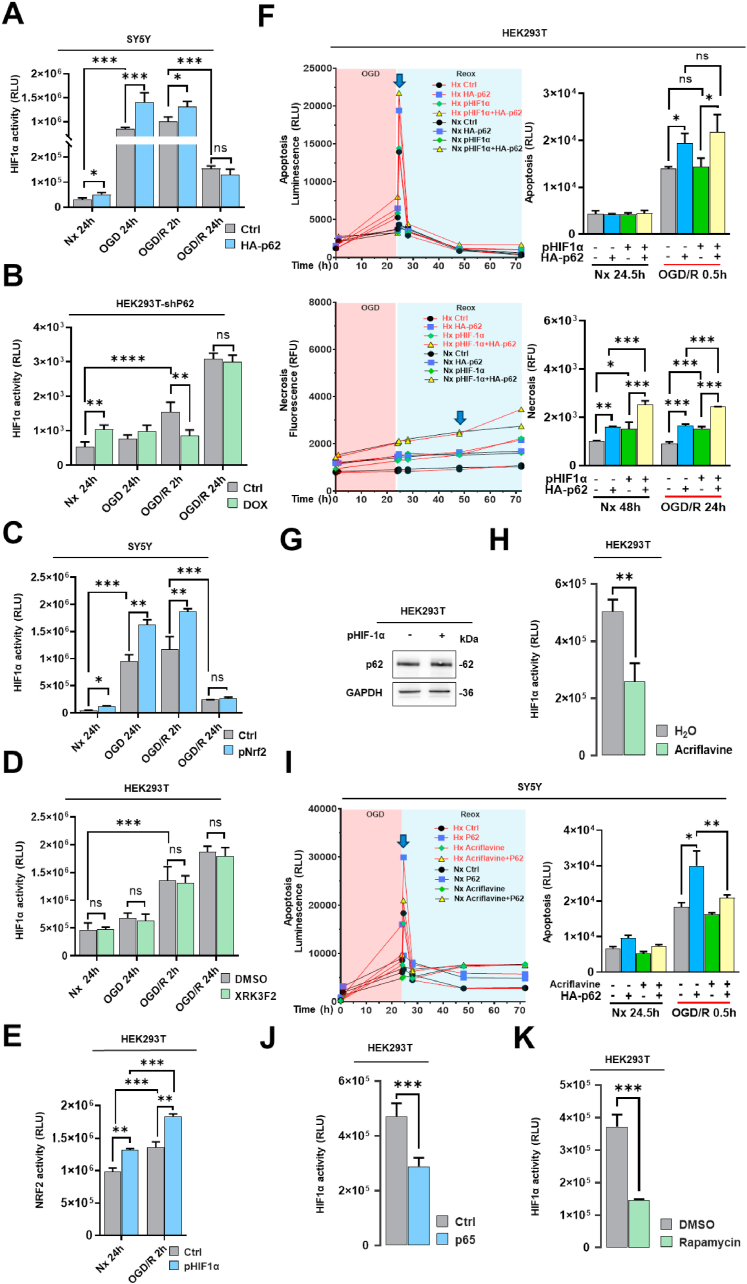
**Roles of HIF1α in mediating p62 signaling responses post-I/R. (A)** HIF1α activity assessed using a hypoxia-response element (HRE)-based firefly luciferase reporter system in SY5Y cells overexpressing HA-p62 plasmid, which were exposed to Nx, OGD or OGD/R. **(B)** After inducing shRNA-mediated p62 KD with doxycycline, HIF1α activity was evaluated in HEK293T cells by the firefly luciferase-based reporter system under Nx, OGD or OGD/R conditions. **(C)** HIF1α activity measured by the firefly luciferase reporter system in SY5Y cells overexpressing stable pNRF2 plasmid under Nx, OGD or OGD/R conditions. **(D)** Likewise, HIF1α activity was examined using the firefly luciferase reporter system in HEK293T cells exposed to the p62-ZZ domain inhibitor XRK3F2 (5 μM) under Nx, OGD or OGD/R conditions. **(E)** Conversely, NRF2 activity was assessed using the firefly luciferase reporter system in HEK293T cells overexpressing stable pHIF1α plasmid under Nx, OGD or OGD/R conditions. **(F)** Real-time apoptosis and necrosis levels of HEK293T cells following overexpression of stable pHIF1α and HA-p62 plasmids under Nx and OGD/R conditions. **(G)** Western blot analysis of p62 protein in HEK293T cells overexpressing stable pHIF1α plasmid. **(H)** HIF1α activity measured using the firefly luciferase reporter system in HEK293T cells treated with the HIF1α dimerization inhibitor acriflavine (5 μM) for 24 h. **(I)** Real-time apoptosis levels of SY5Y cells overexpressing HA-p62 plasmid treated with acriflavine (5 μM) for 24 h, which were exposed to Nx or OGD/R. **(J)** HIF1α activity evaluated by the firefly luciferase reporter system in HEK293T cells overexpressing p65 plasmid. **(K)** HIF1α activity assessed by the firefly luciferase reporter system in HEK293T cells treated with the autophagy inducer rapamycin (1 μM) for 24 h. Plasmid overexpression was initiated 24 h before the study. Plasmid control groups consist of equal amounts of pcDNA3.1. Each experiment was performed three times, with at least three samples processed in the same way in each experiment. Comparisons between two groups were analyzed by Student's t-tests, while data among multiple groups were analyzed using ANOVA followed by Tukey's post hoc tests. Statistical significance was indicated as ∗p < 0.05, ∗∗p < 0.01, ∗∗∗p < 0.001, ∗∗∗∗p < 0.0001.

HIF1α is a key factor mediating cellular responses to Hx, activating the transcription of multiple genes involved in regulating cell growth and apoptosis [33]**.** To investigate a potential interaction of p62 and HIF1α post-I/R, we conducted luciferase-based reporter gene assays and found that p62 overexpression increased, whereas p62 KD deceased HIF1α activity (Fig. 9A and B), probably mediated indirectly through p62-stabilized NRF2 levels (Fig. 7, Fig. 9C). When cells were incubated with p62 inhibitor XRK3F2, it did not significantly influence HIF1α activity (Fig. 9D). Since NRF2 overexpression increased HIF activity (Fig. 9C), we overexpressed a non-degradable HIF1α mutant in HEK293T cells. Interestingly, under both Nx and OGD/R conditions, HIF1α enhanced NRF2 activity (Fig. 9E). This suggests a bidirectional positive feedback loop. We also expressed stable HIF1α in HEK293T cells to detect apoptosis, which did not influence Reox-related apoptosis but increased late-stage (24 h) necrosis during Nx and Reox (Fig. 9F). However, Western blotting showed that overexpressing HIF1α in HEK293T cells for 24 h did not affect p62 protein levels (Fig. 9G). Acriflavine is an inhibitor of HIF1α dimerization, which reduced HIF1α activity in luciferase reporter assays (Fig. 9H). Although acriflavine alone did not reduce apoptosis during early (0.5 h) Reox in HEK293T cells, it reduced apoptosis induced by p62 overexpression (Fig. 9I). We also found that overexpression of the NFκB subunit p65 reduced HIF1α activity in HEK293T cells (Fig. 9J), as did autophagy activation by rapamycin (Fig. 9K).

### 2.10 p62 inhibition reduces infarct volume, neuronal death and the activation of microglia/macrophages via anti-inflammatory mechanisms after ischemic stroke *in vivo*

**Fig. 10:**
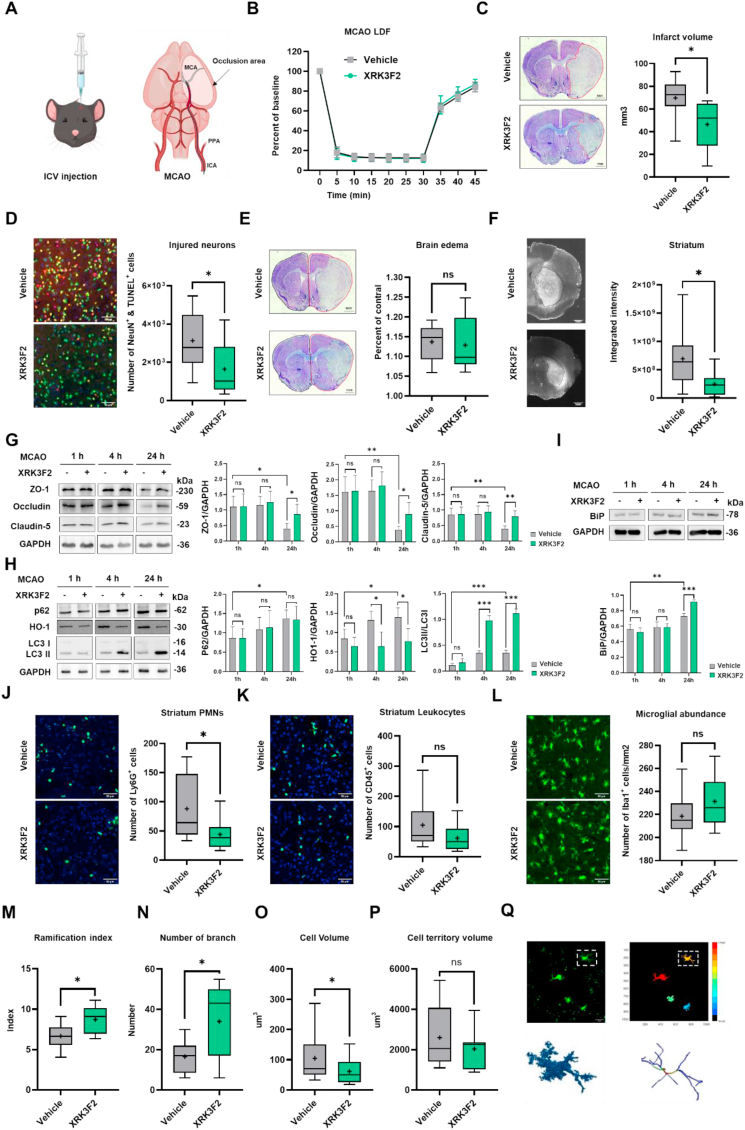
**Pharmacological p62 deactivation induces neuroprotection in a mouse ischemic stroke model. (A)** Schematic outline of the experiment. Vehicle (2 % DMSO) or the p62 inhibitor XRK3F2 (2.5 μg/μL) was injected into the lateral ventricle of mice. Twenty-four hours after middle cerebral artery occlusion (MCAO), brains were collected for cresyl violet staining, immunohistochemistry and Western blot analysis. **(B)** Laser Doppler flow (LDF) measurements above the core region of the middle cerebral artery territory. Note the reproducible decline of blood flow during MCAO, followed by the rapid restoration of blood flow after reperfusion. Recordings did not differ between vehicle and XRK3F2 groups. **(C)** Infarct volume assessed by cresyl violet staining, **(D)** neuronal injury in the ischemic striatum analyzed by NeuN (in green)/TUNEL (in red) double staining, **(E)** brain edema assessed by cresyl violet staining, and **(F)** blood-brain barrier permeability assessed by serum IgG extravasation analysis of mice exposed to MCAO that were sacrificed 24 h after reperfusion (n = 9 animals/group). Western blot analysis of **(G)** zona occludens-1 (ZO1), occludin and claudin-5, **(H)** p62, heme oxygenase-1 (HO1) and LC3-II/I and **(I)** BiP in ischemic brain tissue of MCAO mice treated with vehicle or XRK3F2 which were sacrificed 1, 4 and 24 h after reperfusion (n = 4 animals/group). Blots in (G–I) were normalized with GAPDH loading controls. For LC3 blots LC3-II/I ratios were formed. Brain infiltration of **(J)** Ly6G^+^ polymorphonuclear neutrophils and **(K)** CD45^+^ leukocytes in the ischemic striatum of vehicle or XRK3F2 treated MCAO mice sacrificed 24 h after reperfusion (n = 9/group). Representative immunostaining images are shown. **(L)** Accumulation of Iba1^+^ microglia/macrophages in ischemic striatum of vehicle or XRK3F2 treated MCAO mice sacrificed 24 h after reperfusion (n = 9/group). Representative immunostaining images are shown. Morphology analysis of Iba1^+^ microglia/macrophages in the ischemic striatum revealing microglial **(M)** ramification, **(N)** branch number, **(O)** cell volume, and **(P)** cell territory volume assessed in **(Q)** high-resolution 3D stacks, in which individual microglia/macrophages were segmented, skeletonized and reconstructed (example highlighted in a square in Q) (n = 9/group). Scale bars: 1 mm (in C, E, F), 50 μm (in D, J-L). Data in (B) and (G–I) are mean + SD values. Data in (C–F) and (J–P) are box plots indicating medians (lines inside boxes)/means (crosses inside boxes) ± interquartile ranges, with minimum/maximum values shown as whiskers. Comparisons between two groups were analyzed by Student's t-tests, while data among multiple groups were analyzed using ANOVA followed by Tukey's post hoc tests. Statistical significance was indicated as ∗p < 0.05, ∗∗p < 0.01, ∗∗∗p < 0.001. (A) was created with open source software GDP (Generic Diagraming Platform, https://biogdp.com).

To explore the relevance of these different mechanisms *in vivo* in ischemic stroke, we administered the p62 ZZ domain inhibitor XRK3F2 (2.5 μg/μL) into the lateral ventricle of mice 24 h before transient MCAO (Fig. 10A). Laser Doppler flow (LDF) measurements above the core of the middle cerebral artery (MCA) territory revealed that cerebral blood flow dropped to 10–20 % of baseline during MCAO, followed by the recovery of blood flow to 80–90 % of baseline within 15 min post-MCAO (Fig. 10B), indicating reproducible I/R. Body weight and spleen weight of MCAO mice revealed no differences between vehicle and XRK3F2 groups (Fig. S2A and B). To evaluate whether XRK3F2 treatment provided neuroprotection against I/R damage, we assessed infarct volume by Nissl staining and evaluated the density of DNA-fragmented (i.e., irreversibly injured) neurons in the striatum, which is the core of the MCA territory, by TUNEL (in red) and NeuN (in green) staining 24 h post-MCAO. Infarct volume and the density of TUNEL^+^/NeuN^+^ injured neurons were decreased in XRK3F2 treated compared to vehicle treated MCAO mice (Fig. 10C and D). To examine neurovascular integrity, we next examined brain edema in Nissl stainings and measured blood-brain barrier (BBB) permeability by serum IgG extravasation analysis. These studies revealed that, while brain edema was nominally, but not significantly decreased (Fig. 10E), IgG extravasation was reduced by XRK3F2 (Fig. 10F), indicative of BBB preservation. Next, we assessed the abundance of tight junction proteins in ischemic brain tissue at 1, 4, and 24 h post-MCAO by Western blotting, revealing an increased zona occludens-1 (ZO1), occludin and claudin-5 levels in XRK3F2 treated compared to vehicle treated MCAO mice 24 h post-MCAO (Fig. 10G). All three junctional proteins exhibited a pronounced reduction of more than 50 % in the control group at 24 h post-MCAO, indicating a substantial BBB integrity loss, which was partially rescued by XRK3F2 treatment. p62 abundance in ischemic brain tissue was not influenced by XRK3F2 (Fig. 10H), but LC-II/I ratio was increased 4 and 24 h post MCAO (Fig. 10H), indicative of autophagic flux congestion induced by p62 deactivation. In line with the *in vitro* studies, HO1, the key antioxidant downstream effector protein of NRF2, was reduced by XRK3F2 starting 4 h post-MCAO (Fig. 10H), while the ER stress sensor BiP was increased 24 h post-MCAO (Fig. 10I). To study inflammatory responses, we examined the abundance of intercellular adhesion molecule-1 (ICAM1) on brain endothelial cells, which was not significantly altered by XRK3F2 (Fig. S2C), while the density of brain-invading Ly6G^+^ polymorphonuclear neutrophils (PMNs) and CD45^+^ leukocytes was reduced (Fig. 10J and K), albeit the latter failed to reach statistical significance. Brain-invading PMNs critically exacerbate I/R injury in the stroke recovery phase [34]. The density of Iba1^+^ microglia in ischemic brain tissue was not influenced by XRK3F2 (Fig. 10L), but microglial morphology analysis revealed that 24 h after MCAO, XRK3F2 increased the microglial ramification and branch number (Fig. 10M and N), while reducing the cell volume (Fig. 10O). However, it had no effect on cell territory volume (Fig. 10P), as demonstrated by 3D stacks where individual cells were reconstructed, segmented, and skeletonized for quantitative analysis (Fig. 10Q). Taken together, these results indicate that XRK3F2 inhibited the activation of microglia/macrophages, converting them into a ramified resting state. Our results reveal that p62 inhibition decreased brain damage presumably by rebalancing ROS-associated pro-inflammatory responses.

## 3 Discussion

Through GOF and LOF experiments in two neuron-like cell lines (SY5Y, HEK293T), primary neurons, hCMEC/D3 brain endothelial cells and U-87 MG astroglial-like cells, which we exposed to OGD and Reox *in vitro*, as well as *in vivo* analysis of mice subjected to transient MCAO, a clinically relevant ischemic stroke model, we demonstrate that the autophagic hub protein p62 is upregulated upon I/R and plays a critical role in regulating cell survival and death. Indeed, both endogenously expressed p62 and exogenously overexpressed p62 were found to trigger autophagic flux accompanied by elevated ROS burden, detrimental HIF1α signaling and cell death. At the same time, p62 activated a broad network of cytoprotective responses, which included a bidirectional positive feedback loop of p62 and NRF2-dependent antioxidative responses, a bidirectional negative feedback loop of p62 and the pro-inflammatory NFκB protein, and, as a critical pro-survival mechanism, depletion of the ER stress sensor BiP, activating UPR via the PERK branch. *In vivo*, p62 deactivation using the pharmacological inhibitor XRK3F2 significantly improved stroke outcome revealed by a large set of histopathological readouts. That p62 can activate the NRF2-dependent antioxidant pathway via interaction with KEAP1 [10] and downregulate the ER stress sensor BiP/GRP78 via autophagic degradation [35] has previously been reported. That p62 upregulation can inhibit the pro-inflammatory NFκB pathway is new. Of note, none of the signaling pathways has hitherto been examined under cerebral I/R conditions. Our study provides the first comprehensive analysis of the role of p62 post-I/R, providing a conceptual framework for the interpretation of ambiguous observations on the role of autophagy in I/R injury development after stroke [5,6].

In this study, a bidirectional positive feedback loop between p62 and NRF2 was found during I/R conditions. Admittedly, the molecular mechanism has only been elucidated in one direction, namely from p62 to NRF2 via KEAP1 degradation. However, our observation that NRF2 upregulates p62 protein levels is supported by previous findings identifying a functional ARE within the p62 promoter region [36]. In addition to the oxidative stress burden associated autophagy induction, we identified a self-amplifying NRF2-HIF1α signaling network activated by p62. This fits to earlier findings reporting a) a NRF2-targeted antioxidant response element (ARE) in the promoter region of HIF1α and b) a protein-stabilizing effect of NRF2 on HIF1α [37,38]. On the other hand, HIF-1α supports glucose metabolism that triggers cellular production of ROS [39] which was reported to oxidize several highly reactive cysteines in the ROS sensor and NRF2 repressor KEAP1 and interrupts the weak NRF2-KEAP1 interaction thereby stabilizing NRF2 [40]. We observed that this NRF2-HIF1α signaling loop plays a dual role in stroke settings: First in p62-mediated protection of neuronal cells via NRF2 signaling and secondly in ROS-induced apoptotic cell death since chemical inhibition of HIF1α strongly diminished Reox-induced apoptosis suggesting HIF1α may be involved in detrimental ROS formation. Previous studies identified the ROS-producing NADPH oxidases NOX2 and NOX4 as direct HIF1α targets [41,42]. NOX2 and NOX4 were found to be expressed in neurons, astrocytes, microglia and cerebral microvessels [43,44]. In our hands, we did not find any Reox-associated changes in NOX2 and NOX4 levels that could explain this HIF1α-mediated cell death. Therefore, we further tested for BNIP3, another HIF1α target which was shown to be involved in a caspase-independent apoptotic pathway via mitochondrial release of AIF and Endo G [45]. Indeed, we observed a strong upregulation of BNIP3 when p62 overexpression was combined with OGD/R conditions. Although this finding partly confirmed an earlier study in the human tubular cell line HK2, in which HIF1α induced BNIP3 expression after I/R injury [46], the outcome was quite different. While we observed a mitophagy-independent, p62-mediated pro-apoptotic HIF1α-BNIP3 signaling, the authors showed a HIF1α-BNIP3 mediated cell-protective mitophagy response. The dual function of BNIP3, promoting apoptosis by overcoming BCL2-mediated suppression via its BH3 domain and supporting cell survival through LC3-mediated mitophagy via its LIR motif, may be shifted toward one pathway over the other, depending on the cell type. Neurons are highly dependent on mitochondria for ATP production. Consequently, they permit only minimal levels of mitophagy, even under severe stress such as I/R injury [47]. This supports our finding that BNIP3 predominantly induces an apoptotic rather than a mitophagic response in neurons. When monitoring autophagy, it is critical to emphasize that LC3-II protein levels, like LC3-positive fluorescent puncta, reflect autophagosome abundance at a specific time point and do not represent the complete autophagic process, which culminates in cargo degradation within autolysosomes [48]. In our p62 GOF experiments, although autophagic flux was comparable between the two neuron-like cell lines, Western blot analysis revealed a significantly higher LC3-II/I ratio in SY5Y compared to HEK293T cells. Given that p62 is prone to aggregation [49], overexpressed p62 may form more insoluble aggregates in HEK293T cells, potentially due to less stringent proteostatic regulation. This could reduce p62's autophagy-promoting activity and LC3-II accumulation without affecting overall autophagic turnover.

It is noteworthy that we did not detect relevant levels of caspase-3 activity accompanying I/R induced cell death. Although we confirmed the appearance of necroptosis during the Reox phase, its impact on cell death was only minor. Neither was ferroptosis involved, described as a type of regulated necrosis [50], since we were not able to detect any plasma membrane rupture during early Reox [51]. Interestingly, a new caspase-independent apoptosis-like cell death pathway was discovered called oxeiptosis, which was characterized by high ROS levels and NRF2 signaling, which reflects our observations. NRF2 is the key player in the antioxidant response and therefore considered as strongly cell-protective, but was postulated to become an essential part of the pro-apoptotic oxeiptosis signaling in the context of excessive ROS production [52]. In line with a former study was our observation of a dramatic p62-induced upregulation of autophagy without a concomitant mitophagic increase [53]. Therein, the authors found that p62 mediates the aggregation of dysfunctional mitochondria but does not promote cell-protective mitophagy. Since it has also been demonstrated before that oxidative stress induces autophagic cell death under I/R conditions [54], we prefer to argue that the observed p62-triggered cell death in the early Reox phase is a mixed form of caspase-independent programmed cell death consisting of predominantly p62-dependent ROS-induced apoptosis/oxeiptosis, autophagic cell death and to a minor extent perhaps necroptosis.

Of note, previous studies reported diverse effects of p62 on cell death in other disease settings, which may partly be attributed to cell type-specific roles of NFκB. For instance, an *in vitro* cancer study found that p62 silencing induced caspase-3 independent autophagic cell death [55], whereas its upregulation was reported to activate a survival mechanism via NFκB that prolonged the life span of mature acute myeloid leukemia (AML) cells [56]. In our study, overexpression of p62 clearly disrupted NFκB signaling, thereby sensitizing cells to apoptotic signals. One possible mechanism involves sequestration of TRAF6 by the TB domain of p62, promoting its autophagic degradation and thereby attenuating NFκB activity. This was the case in a previous report, where the authors confirmed a downregulation of NFκB target genes by p62 GOF experiments [57]. A competition for binding partners would also be conceivable between p62 and proteins involved in NFκB signaling. For instance, p62 might compete with NFκB essential modulator (NEMO) for binding to ubiquitinated proteins, thereby disrupting the formation of the IKK (IκB kinase) complex necessary for NFκB activation [58]. We were further able to show that the inhibitory effect between p62 and NFκB was a bidirectional event indicating a NFκB-induced inhibition of p62 signaling. This may be a neuronal cell type-specific phenomenon, as a previous study in macrophages demonstrated that NFκB activity actually promoted p62-mediated removal of damaged mitochondria thereby limiting its own inflammation-promoting activity [59]. NFκB's effects on inflammatory responses are strongly context-dependent. NFκB plays an important role in the activation of innate immune responses, having both positive and negative transcriptional regulatory roles [60].

That the ER stress sensor BiP/GRP78 is downregulated by p62 was also found in a former study in which N-arginylated BiP, modified by the N-end rule pathway and relocalized to the cytoplasm, bound the ZZ domain of p62 and got degraded via autophagy [35]. BiP deprivation was shown to have far-reaching consequences in neuronal cells, where it leads to increased oxidative stress and a compromised calcium balance [61], at the same time controlling the activity of all UPR branches, which is a rescue mechanism to regain ER homeostasis with direct impact on the functionality of mitochondria [62]. Since our BiP rescue experiments demonstrated a devastating increase of the apoptotic peak after OGD/R, we rather conclude that loss of BiP after p62 upregulation is not responsible for the additional ROS accumulation in the Reox phase but instead provides protection through activation of the UPR. Taken together, we herein propose a dual role of p62 after OGD/R with a greater adverse effect via ROS-associated oxidative burden that results in cell growth inhibition, apoptotic and autophagic cell death and a subordinate cell-protective effect via counter-regulatory antioxidant responses, anti-inflammation and UPR activation. *In vivo*, the pharmacological inhibition of p62 protected against cerebral I/R injury post-MCAO, increased blood-brain barrier integrity and reduced neuroinflammation evidenced by reduced microglial activation and reduced brain neutrophil infiltration.

**Fig. 11:**
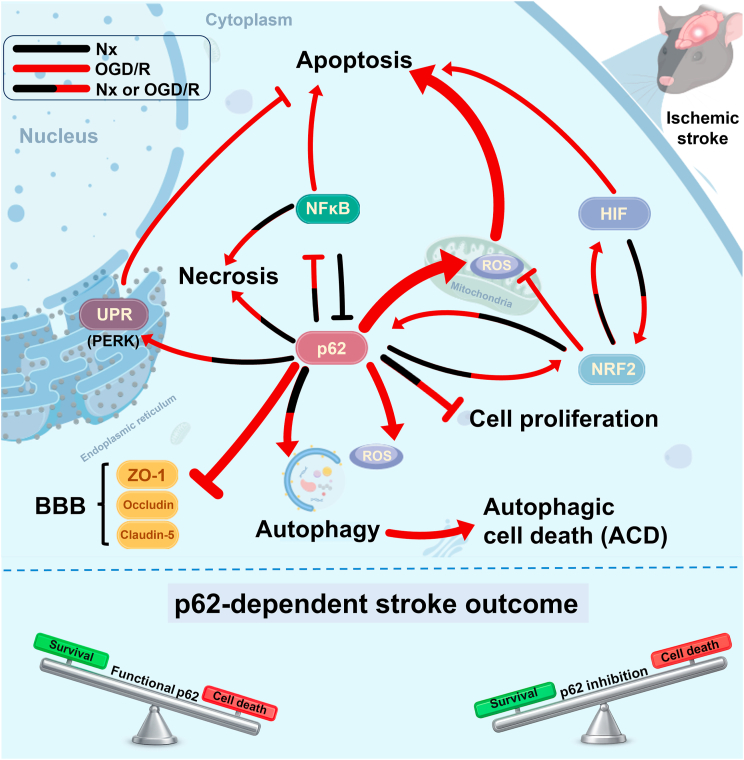
**Signaling processes orchestrating effects of p62 protein on cerebral I/R injury.** p62 was found to influence I/R injury in multiple ways, namely via [1] autophagy-associated ROS formation that results in autophagic cell death [2], a bidirectional activating crosstalk of p62 with NRF2-dependent oxidative stress responses that fosters the activity of HIF1α [3], a bidirectional inhibitory crosstalk of p62 with the transcription factor NFκB, and [4] a unidirectional activation of unfolded protein responses by p62 via depletion of the ER stress sensor BiP and consecutive activation of the UPR PERK branch. The latter signal pathways were shown to control apoptotic and necrotic cell death processes. Crosstalk between key nodal molecules is illustrated for normoxia and/or oxygen-glucose deprivation/reoxygenation in different colors (black for Nx, red for OGD/R). Arrows represent activating interactions, blunt-ended lines denote inhibition. The varying thickness of arrows/blunt-ended lines indicates the relative strength of the effects. The balance scales at the bottom of the figure depict how p62 inhibition alleviates its detrimental impact during stroke. The schematic illustration was created with open source software GDP (Generic Diagraming Platform, https://biogdp.com).

The present study provides a detailed analysis of the role of the autophagy hub protein p62 under cerebral I/R conditions. Various molecular interactions of p62 already described in previous studies could be confirmed and new interactions, such as the self-amplifying NRF2-HIF1α signaling network activated by p62 or the p62-mediated pro-apoptotic HIF1α-BNIP3 signaling response, could be identified. The major findings of this study are summarized in Fig. 11. For sure, in this study not all signaling networks could be evaluated at similar depth. Some signaling interactions will have to be explored in future studies.

Previous studies suggested p62 as a potential target for clinical application, especially for cancer treatment [63]. However, to the best of our knowledge, there are no clinical trials to date that have tested the efficacy of p62 modulators in ischemic stroke. In GOF and LOF experiments we revealed that p62-associated autophagy is a major oxidative stress burden and that p62 deactivation protects against ischemic stroke. Based on our findings, p62 offers itself as a potential target for stroke therapy. A limitation of our *in vivo* experiments is the exclusive use of young male mice, which was intended to reduce variability associated with hormonal cycles in female mice, but also precludes assessment of potential sex-specific effects on stroke outcomes after treatment with XRK3F2. Future studies will have to examine aged and female mice, as well as ischemic mice with vascular risk factors. The complexity of signaling actions induced by p62 emphasizes the need of comprehensive mechanistic studies, which provide thorough insights into signaling networks. Without such studies, premature clinical trials on other oxidative stress targets will carry risks of side effects or study failures. Unsuccessful clinical stroke trials of the past on antioxidant therapies [64] exemplify these risks. As outlined above, p62 has multiple roles in cellular homeostasis, and its inhibition may potentially lead to unforeseen consequences in cellular functions that go beyond I/R injury. Yet, with recent progress in the development of recanalization therapies (i.e., thrombolysis and thrombectomy) [65,66], there meanwhile is a robust correlate of reperfused brain tissue in ischemic stroke patients, which is predisposed to I/R injury, unless oxidative damage is prevented by specifically tailored therapies. In view of these recent clinical advances, this study provides the strong claim that it is time to re-evaluate oxidative stress responses in cerebral I/R injury. Considering their potent impact on stroke outcome, we predict that strategies aiming at reregulating cellular stress responses may guide stroke therapies to new breakthroughs.

## 4 Material and methods

### 4.1 Antibodies and reagents

Antibodies against NQO1, PARP, GAPDH, p-p62, CAS-3, LC3, GAPDH, BiP, ZO1 and phospho-eIF2α were purchased from Cell Signaling (Frankfurt/Main, Germany). NOX2, NOX4, NRF2 and p62 antibodies were from Proteintech (Munich, Germany). KEAP1, ATF4, HO-1, BNIP3 and occludin antibodies were from Abcam (Cambridge, UK). Claudin-5 antibody was from Santa Cruz Biotechnology (Dallas, USA). HRP-coupled secondary anti-rabbit and anti-mouse antibodies were from Dako (Hamburg, Germany). Chemical compounds were obtained from Sigma (Munich, Germany), except for the p62-ZZ/domain inhibitor XRK3F2 and the receptor-interacting protein kinase-1 (RIPK1) blocker necrostatin-1, an inhibitor of necroptosis, which were from MedChemExpress (NJ, USA).

### 4.2 Plasmids

If not stated otherwise, the following plasmids were purchased from Addgene Repository (http://www.addgene.org) and used for transient transfections: pGL3-8xARE, HA-p62 (#28027), Flag-NRF2 (#36971), Flag-KEAP1 (#28023), pRluc-LC3-WT (#105002), pRluc-LC3-G120A (#105003), 4xNFκB-Luc (#111216), pCDNA3-FLAG TRAF6 (#66929), eGFP-p65 (#111190), 3xHRE-Luc (#118706), p(HA)HIF1α (P402A, P564A) (#52636), mcherry-p62-WT (#187986), mcherry-p62-Δ-UBA(#187984), mcherry-p62-Δ-PB1 (#187985), mcherry-p62-Δ-LIR (#187981), mKeima-Red-Mito-7 (# 56018). Unless otherwise stated, pcDNA3.1 (#138209) or pEGFP-N1 (#172281) were used as control.

### 4.3 Cell culture and transfection

HEK293T, SH-SY5Y (hereafter SY5Y) and U-87 MG cells were cultured in high-glucose Dulbecco's Modified Eagle Medium (DMEM; Invitrogen, Darmstadt, Germany) supplemented with fetal bovine serum (FBS; 10 % for HEK293T and U-87 MG, 12 % for SY5Y) and 1 % penicillin/streptomycin. Human cerebral microvascular endothelial hCMEC/D3 cells were cultivated from passage 28 to 35 in Growth Basal Medium-2 (EBM-2, Lonza, Basel, Schweiz) supplemented with 5 % fetal FBS, 1 % penicillin/streptomycin, 1.4 μM hydrocortisone (Sigma-Aldrich, Saint Louis, U.S.A.), 5 μg/ml ascorbic acid (Sigma-Aldrich), 1 % chemically defined lipid concentrate (Life Technologies), 10 mM HEPES (Life Technologies) and 1 ng/ml basic fibroblast growth factor (bFGF, Sigma-Aldrich). Mouse primary neurons were prepared as described before [67]. For normoxic (Nx) and reoxygenation (Reox) conditions, cells were incubated with 21 % O_2_, 5 % CO_2_ and 25 mM glucose. For oxygen-deprived conditions, cells were kept in a hypoxic chamber (1 % O_2_, 5 % CO_2_, Toepffer Lab System, Göppingen, Germany) with either 25 mM glucose (Hx) or without glucose (OGD). Transient transfections were performed with lipofectamine 3000 transfection reagent (Thermo Fisher Scientific, Oberhausen, Germany) following the manufacturer's instructions. All experiments were performed with mycoplasma-free cells.

### 4.4 PiggyBac-based knockdown of p62

HEK293T cells were co-transfected with pPB-TetOn-shp62 vector (VB220727-1145xvw, Vector Builder Inc, USA) and a PiggyBac transposase-expressing plasmid (VB010000-9365tax, Vector Builder Inc, USA) using transfection reagent lipofectamine 3000. After 48 h, selection was conducted for 1–2 weeks using 1 μg/mL puromycin to enrich cells that have integrated the PiggyBac transposon. For knockdown (KD) experiments, three different shRNAs (shRNA-1: 5ʹ-CGCAGATGAGAAAGATCGCCTT-3ʹ; shRNA-2 5ʹ-CCCGAATCTACATTAAAGAGA A-3ʹ; shRNA-3: 5ʹ-ACCTCTGGGCATTGAAGTTGAT-3ʹ) against p62 mRNA (GenBank acc. no. NM_001142298) were expressed by addition of 250 ng/ml doxycycline to the growth medium.

### 4.5 Luciferase-based gene reporter assay

We measured NRF2, NFkB and HIF transcription activity by performing Luciferase reporter gene assays: 6 × 10^4^ SY5Y or 3 × 10^4^ HEK293T cells were seeded in 96-well plates. Next day cells were transfected with 50 ng of pGL3-8xARE (NRF2 activity), pGLHIF1.3 (HIF activity) or 4xNFκB-Luc (NFκB activity). For p62 GOF experiments, cells were co-transfected with 100 ng of plasmid HA-p62 and control plasmid pcDNA3.1. Cell lysis was done by using passive lysing buffer (Promega, Madison, USA). Firefly luciferase were measured using the multi-mode microplate reader Synergy™ HT (BioTek, Bad Friedrichshall, Germany) and normalized to cell numbers.

### 4.6 Mitophagy detection by flow cytometry

For detection of mitophagic activity, 2 μg of plasmid mKeima-Red-Mito-7 was transfected into 1 × 10^6^ pre-seeded cells using lipofectamine 3000 (Thermo Fisher Scientific). For p62 GOF tests, co-transfection was conducted with 1 μg of HA-p62 and control plasmid pcDNA3.1. A red laser (633 nm) coupled with a 660/20 nm bandpass filter (APC channel) was used to detect the excitation switch due to a pH drop of as the last step of autophagy. At least 20.000 cells per sample were analyzed on a LSRII (BD Biosciences, Franklin Lakes, NJ, USA) using FACS DIVA software.

### 4.7 Apoptosis and necrosis assay

The RealTime-Glo™ Annexin V Apoptosis and Necrosis Assay (Promega, Madison, WI, USA) was utilized following manufacturer guidelines. This assay detects phosphatidylserine exposure during apoptosis, with annexin V binding detected through luminescence and necrosis through fluorescence signals, using a plate-based multimode reader. For the experiments, cells were plated in a black 96-well assay plate after 48 h of culture in complete medium, as per the manufacturer's instructions. At specified time points (0, 24, 24.5, 28, 48, and 72 h), the luminescence and fluorescence (excitation: 460 nm and emission: 528 nm) was recorded using the multi-mode microplate reader Synergy™ HT (BioTek, Bad Friedrichshall, Germany).

### 4.8 Cell proliferation assay

Cell proliferation was assessed following the instructions. Briefly, 1.8 × 10^5^ HEK293T cells or 3.6 × 10^5^ SY5Y cells were plated in a 24-well plate and subsequently the KD was induced. After incubation for 24h in Hx, cells were fixed with 4 % PFA. Next, cells were washed with 70 % ethanol and incubated with Coomassie brilliant blue. Finally, the colored reaction indicates cell proliferation was quantified analysis software ImageJ.

### 4.9 Cell cycle analysis

Distinct phases of the cell cycle were distinguished by DNA staining with the fluorescent dye propidium iodide and measured by flow cytometry. Cells were washed in ice cold 0.1 M phosphate-buffered saline (PBS), and stained for 30 min at 37 °C with the fluorescent dye propidium iodide followed by flow cytometric analysis. The percentages of cells in G1, S, and G2/M phases were determined using the BD LSR II instrument (BD Biosciences, San Jose, CA, USA).

### 4.10 Autophagic flux reporter assay

Autophagic flux was assessed using a Renilla luciferase-based reporter assay as previously described [68]. Briefly, cells were co-transfected with Rluc-LC3wt and Rluc-LC3G120A plasmids and seeded into black 96-well plates. Six hours post-transfection, the medium was replaced. Prior to measurement, 60 μM Viviren substrate (Promega, Madison, WI, USA) was added to the medium. Rluc-LC3 activity was dynamically monitored using a Synergy™ HT multi-mode microplate reader (BioTek, Bad Friedrichshall, Germany) over a 24-h period. Autophagic flux was quantified as the ratio of Rluc-LC3mut to Rluc-LC3wt luminescence intensity.

### 4.11 Transmission electron microscopy (TEM)

For TEM analysis, HEK293T cells were cultured in glass-bottom dishes (μ-Dish 35 mm high Grid-500; Ibidi GmbH, Gräfelfing, Germany). At the timepoint of analysis, the cells were fixed using 4 % formaldehyde +2.5 % glutaraldehyde dissolved in 0.1 M PHEM buffer, overnight, at 4 °C. All following steps of the sample preparation, including post-fixation with osmium tetroxide, contrasting with uranyl acetate, dehydration in an ascending ethanol row and EPON™ (Poly/Bed 812, Polysciences Inc., Warrington USA) infiltration were performed using a laboratory microwave oven (PELCO BioWave Pro+, Ted Pella, Redding, USA). A table containing the microwave protocol details can be found in the supplemental information (Table S1). The resin polymerization then took place at 60 °C for 96 h. After subsequent glass bottom removal and release from the dish the resin disks were trimmed to areas of interest. Ultrathin sections of 100 nm in diameter were cut using an ultramicrotome (EM UC7, Leica Microsystems, Wetzlar, Germany) equipped with a diamond knife (ultra 35°, Diatome, Nidau, Switzerland). Ribbons of sections were collected on 10 mm × 10 mm pieces of silicon wafer (4″ diced Silicon Wafer, Ted Pella, Redding, USA). For better adhesion the sections were baked at 60 °C for 30 min on the silicon substrate. On-section staining was done with 4 % aqueous uranyl acetate for 10 min and Reynold's lead citrate solution for 6 min. Each step followed by extensive washing with distilled water. Image acquisition was performed with a Zeiss Crossbeam 540 (Carl Zeiss Microscopy Deutschland GmbH, Oberkochen, Germany) equipped with a back scattered electron detector (SenseBSD, Carl Zeiss Microscopy Deutschland GmbH, Oberkochen, Germany) using the Zeiss Atlas 5 software (v.5.3.5.3) for large area mappings and high resolution images. High tension of the electron beam was set to 2 kV with a beam current of 1 nA. Working distance of the objective lense was 4 mm and the dwell time for high resolution images was set to 4.5 μs with a pixel size of 4 nm.

### 4.12 CellROX and MitoSOX

Oxidative stress measurements were evaluated using CellROX Green Reagent (Thermo Fisher). In all studies, 8 × 10^5^ HEK293T cells were plated. After 1 day under Nx or OGD/R conditions (1 % O_2_), cells were incubated with 5 μM CellROX for 2 h. The CellROX itself is non-fluorescent, but if it gets oxidized in cells, the excitation at 488 nm leads to the emission of intense green fluorescence with wavelengths around 520 nm. The fluorescence was detected using an AMG EVOS fluorescence microscope with a 20× CFI Plan Fluor objective (Thermo Fisher Scientific, Waltham, USA). Similarly, mitochondrial superoxide oxidation by the MitoSOX (Thermo Fisher) reagent results in a bright green fluorescence (510 nm). After incubating HEK293T cells with 1 μM MitoSOX for 2 h, observations were made using a Leica TCS SP8 fully automated epifluorescence confocal microscope (Zeiss, Wetzlar, Germany) at 40× magnification. Fluorescence images were quantified with the image processing and analysis software ImageJ.

### 4.13 Middle cerebral artery occlusion (MCAO), experimental design, and animal grouping

Animal experiments were conducted with approval of the local government (Bezirksregierung Düsseldorf) in strict accordance with the EU Directive 2010/63 on the care and use of laboratory animals, as well as the STAIR, STEPS, and ARRIVE guidelines. To ensure unbiased group allocation, animals were randomly assigned to treatment groups using a computer-generated random number sequence prior to the start of the experiment. Rigorous randomization was applied throughout the experimental process. The researcher conducting the animal experiments and histochemical studies (CW) was fully blinded at all stages by another researcher (NH), who was responsible for preparing the vehicle and XRK3F2 solutions. These solutions were labeled as Solution A and Solution B, with the identities revealed only after the study concluded. Animals were housed in groups of 4–5 per cage under a 12-h light/12-h dark cycle, with free access to water and food. All surgical procedures were scheduled for the morning throughout the study.

Male C57BL6/j mice (8–10 weeks old, Harlan Laboratories, Darmstadt, Germany) were randomly divided into two groups: a vehicle group (2 % dimethyl sulfoxide (DMSO)) and an XRK3F2 treatment group (2.5 μg/μL in 2 % DMSO). The administration method and dosage of XRK3F2 refer to those described in previous literature [69]. One day prior to the MCAO procedure, mice were anesthetized with 1.0–1.5 % isoflurane (30 % O_2_, remainder N_2_O) and positioned in a stereotaxic frame. The coordinates for lateral ventricle injection were 0.4 mm posterior to the bregma, 1.0 mm lateral to the midline, and 2.5 mm below the skull surface. Using a Hamilton microsyringe, 2 μL of the vehicle or XRK3F2 solution was injected into the left ventricle at a rate of 1 μL/min. After the injection, the microsyringe was left in place for 10 min to minimize reflux.

Mice were anesthetized with 1.0–1.5 % isoflurane (30 % O_2_, balance N_2_O) and subjected to 30 min left-sided intraluminal MCAO. Rectal temperature was maintained between 36.5 °C and 37.0 °C using a feedback-controlled heating system (Fluovac, Harvard Apparatus, Holliston, MA, USA). Cerebral blood flow was monitored via laser Doppler flowmetry (LDF) using a flexible probe attached to the skull above the core of the middle cerebral artery territory. The left common carotid artery and external carotid artery were isolated and ligated, and the internal carotid artery was temporarily clamped. A silicone-coated nylon filament (Doccol Corp., Sharon, MA, USA) was introduced through a small incision into the common carotid artery and advanced to the carotid bifurcation for MCAO. Reperfusion was initiated 30 min later by filament removal. The surgical wound was carefully sutured. Throughout the study, all mice received buprenorphine (0.1 mg/kg, Reckitt Benckiser, Slough, UK) for analgesia. Post-operatively, animals received intraperitoneal injections of carprofen (5 mg/kg, Bayer Vital, Leverkusen, Germany) every 12 h to reduce inflammation.

One day after MCAO, mice (n = 9 per group) were deeply anesthetized and euthanized by transcardial perfusion with 0.1 M PBS followed by 0.1 M PBS containing 4 % paraformaldehyde for immunohistochemistry. Another group of mice (n = 4 per group) was deeply anesthetized and euthanized by transcardial perfusion with 0.1 M PBS for Western blot analysis.

### 4.14 Infarct volume and brain edema assessment

Coronal cryostat sections of the forebrain, 20 μm thick, were collected at 1 mm intervals and stained with cresyl violet. The sections were scanned and analyzed using ImageJ software (National Institutes of Health, Bethesda, MD, USA). Infarct volume was determined by subtracting the areas of healthy tissue in the ischemic hemisphere from those in the contralateral hemisphere at various brain levels. Brain edema was assessed by evaluating the increase in volume of the ipsilateral hemisphere compared to the contralateral hemisphere.

### 4.15 Immunohistochemistry

Cryostat sections from the rostrocaudal level of the mid-striatum, which represents the core of the middle cerebral artery territory, were immersed in a 0.1 M PBS solution containing 0.1 % Triton X-100 and 10 % normal donkey serum (D9663; Sigma-Aldrich). The sections were incubated overnight at 4 °C with Alexa Fluor-594-conjugated polyclonal donkey anti-IgG (A-21203; Thermo Fisher Scientific, Waltham, MA, USA), monoclonal rabbit anti-NeuN (neuronal nuclei antigen; ab177487; Abcam), polyclonal goat anti-intercellular adhesion molecule-1 (ICAM1) (AF796; R&D Systems, Minneapolis, MN, USA), monoclonal rat anti-CD45 (05–1416, Merck Millipore), monoclonal rat anti-Ly6G (lymphocyte antigen 6 complex, locus G; 1A8; 127602; BioLegend, San Diego, CA, USA), and polyclonal rabbit anti-ionized calcium binding adapter molecule 1 (Iba1) (019–19741; Wako Chemicals, Neuss, Germany) antibodies. Detection of non-conjugated antibodies was carried out using secondary antibodies conjugated to Alexa Fluor-488, Alexa Fluor-594, or Alexa Fluor-647. Nuclei were counterstained with Hoechst-33342. For NeuN staining, irreversible DNA fragmentation was detected by terminal deoxynucleotidyl transferase dUTP nick end labeling (TUNEL) using a kit according to the manufacturer's instructions (Roche Diagnostics, Mannheim, Germany). The sections were evaluated using a Zeiss AxioObserver.Z1 inverted fluorescence microscope equipped with Apotome optical sectioning. Signal intensity in the striatum (extravascular IgG, ICAM-1) was analyzed, and immunopositive cells in the ischemic striatum (NeuN, TUNEL, NeuN/TUNEL, Ly6G, CD45, Iba-1) were counted. Cell counts were corrected for optical sectioning to avoid duplicate counting.

### 4.16 Microglia/macrophage morphology analysis

Confocal microscopy using the Leica SP8 (63x/1.30 objective, Leica Microsystems, Wetzlar, Germany) was performed to assess the 3D morphology of Iba1^+^ microglia/macrophages in the ischemic striatum. Z-stacks (184.52 × 184.52 × 15 μm, 0.5 μm interslice distance) were analyzed using the MATLAB-based 3DMorph program. Cells were identified via automated thresholding and segmentation, and morphological metrics such as cell territory, volume, and ramification index (territory/volume) were calculated after skeletonization [70]. This workflow uniquely enables precise quantification of individual microglia/macrophages in densely packed brain lesions.

### 4.17 SDS gel electrophoresis (SDS-PAGE) and Western blot

After brain tissue or cells lysis in RIPA buffer (50 mM Tris pH 7.5, 2 mM EDTA, 150 mM NaCl, 1 % Nonidet P40, 0.1 % SDS, 0.5 % sodium desoxycholate), including protease/phosphatase inhibitor cocktail (P5726, Merck KGaA, Darmstadt, Germany), proteins were separated by SDS-PAGE and transferred to polyvinylidene fluoride membrane via Trans-Blot Turbo Blotting System (Bio Rad, California, USA). After blocking the membrane with 5 % BSA in TBS-T (50 mM Tris/HCl, 150 mM NaCl, 0.5 % Tween-20, pH 7.2) for 1 h, antibody incubation of the membrane was conducted as recommended by the manufacturer. Proteins were detected by using an ECL kit (#34096, Thermo Fisher Scientific, Waltham, USA) by a ChemoCam chemoluminescence documentation system (Intas, Göttingen, Germany).

### 4.18 Statistical analysis

Animal statistical planning was conducted a priori using a sample size calculator (https://www.dssresearch.com/resources/calculators/sample-size-calculator-average/). The calculation determined that for histochemical readouts, 9 animals per group would be required. This calculation was based on the assumption that XRK3F2 would produce a 30 % change in the mean value, with the standard deviation of the data set at 25 % of the mean (effect size: 1.2). The alpha error was set at 5 %, and the beta error (1 - statistical power) was set at 20 %.

All *in vitro* results were confirmed in at least three independent experiments with three samples each that were simultaneously processed. Bar graphs represent mean + standard deviation (SD) values. Histochemical analyses of mouse tissues were shown as box plots indicating medians (lines inside boxes)/means (crosses inside boxes) ± interquartile ranges, with minimum/maximum values shown as whiskers. Comparisons between two groups were performed by Student's t-tests. For multiple group comparisons, one-way or two-way ANOVA was used as adequate, followed by Tukey post-hoc test using Prism software (GraphPad Software, Inc., La Jolla, CA, USA). In all figures, ns indicates p > 0.05, ∗indicates p < 0.05, ∗∗indicates p < 0.01, ∗∗∗indicates p < 0.001, and ∗∗∗∗indicates p < 0.0001.

## CRediT authorship contribution statement

**Xingyun Quan:** Writing – review & editing, Writing – original draft, Investigation, Formal analysis, Data curation. **Yukun Yang:** Writing – review & editing, Writing – original draft, Investigation, Formal analysis, Data curation. **Xiaolong Liu:** Writing – review & editing, Writing – original draft, Methodology, Investigation, Data curation. **Britta Kaltwasser:** Validation, Methodology, Investigation. **Matthias Pillath-Eilers:** Methodology, Investigation, Formal analysis. **Bernd Walkenfort:** Methodology, Investigation, Formal analysis. **Sylvia Voortmann:** Methodology, Investigation. **Ayan Mohamud Yusuf:** Writing – review & editing, Validation, Methodology, Investigation. **Nina Hagemann:** Writing – review & editing, Methodology, Investigation. **Chen Wang:** Writing – review & editing, Visualization, Formal analysis, Data curation. **Mike Hasenberg:** Writing – review & editing, Validation, Investigation, Formal analysis. **Dirk M. Hermann:** Writing – review & editing, Writing – original draft, Supervision, Project administration, Investigation, Formal analysis, Data curation, Conceptualization. **Ulf Brockmeier:** Writing – review & editing, Writing – original draft, Validation, Supervision, Project administration, Methodology, Investigation, Formal analysis, Conceptualization.

## Declaration of competing interest

The authors declare no financial, personal, or professional conflicts of interest.

## Data Availability

Data will be made available on request.
