# Supplementary material for: Autophagy hub-protein p62 orchestrates oxidative, endoplasmic reticulum stress, and inflammatory responses post-ischemia, exacerbating stroke outcome
Source: Redox Biol. 2025 May 27;84:103700. doi: 10.1016/j.redox.2025.103700 (PMC12167074; doi:10.1016/j.redox.2025.103700)

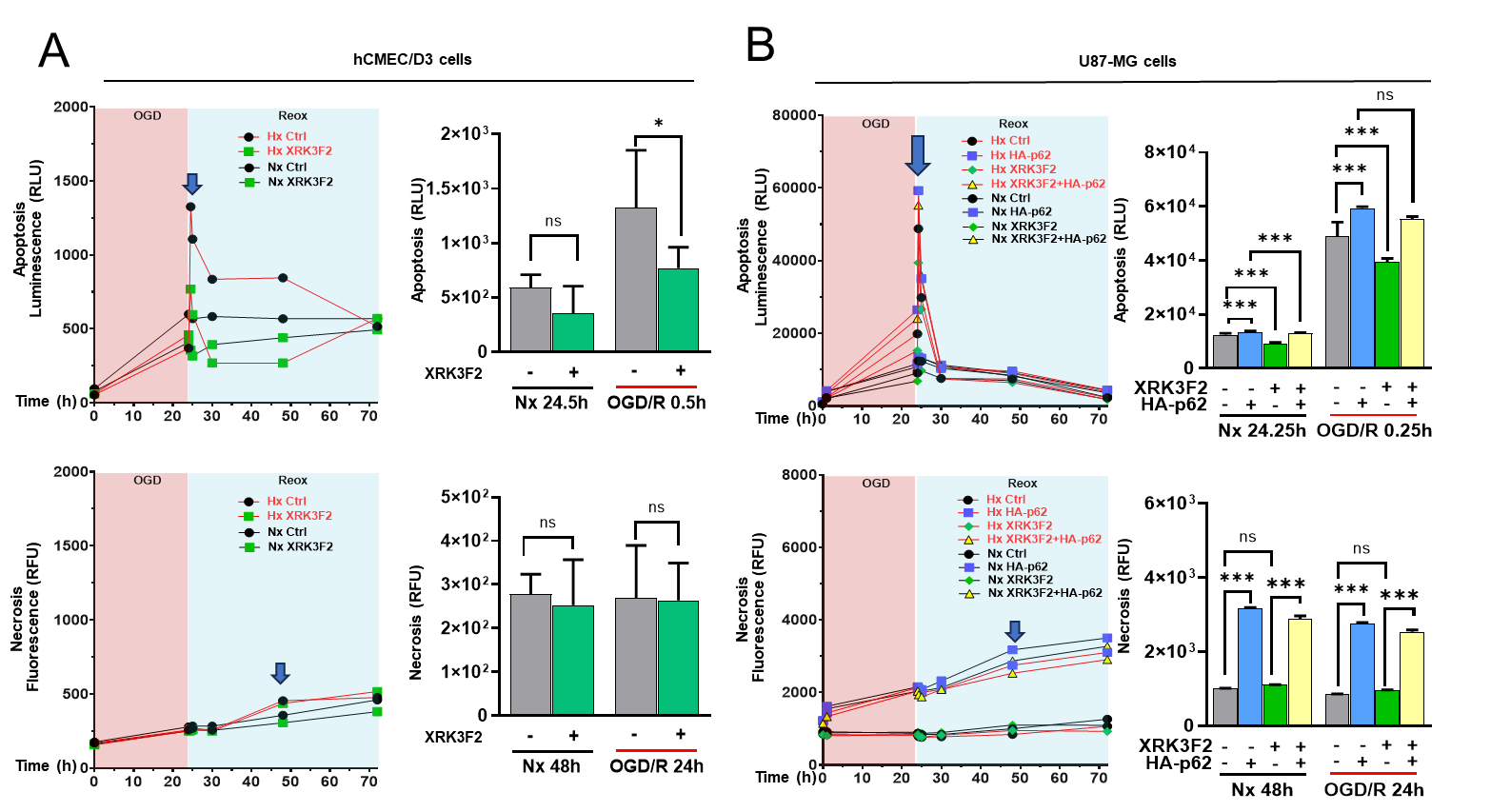


**Fig S1. Analysis of apoptosis and necrosis levels of live hCMEC/D3 brain endothelial and U87-MG astroglial-like cells. (A)** Apoptosis and necrosis levels of live hCMEC/D3 cells measured by the RealTime-Glo Annexin V Apoptosis and Necrosis Assay. Cells were treated with the p62-ZZ domain inhibitor XRK3F2 at 5 μM and placed in a normoxia (Nx; 21% O2) or hypoxia (Hx; 1% O2) environment in regular glucose or glucose deprivation (ΔG) conditions for 24 hours. Bar graphs display the levels of apoptosis after 30 minutes Reox and of necrosis after 24 hours Reox. **(B)** Apoptosis and necrosis levels of live U87-MG cells overexpressing HA-p62 plasmid, which were treated with the p62-ZZ domain inhibitor XRK3F2 at 5 μM during Nx and oxygen-glucose deprivation followed by reoxygenation/ glucose resupplementation (OGD/R). HA-p62 plasmid overexpression was induced 24 hours before the study. Equal amounts of DMSO (2%) and pcDNA3.1 plasmid were used as control groups for XRK3F2 and HA-p62 plasmid, respectively. Each experiment was performed three times, with at least three samples processed the same way in each experiment. Data are mean + SD values. Statistical comparisons were performed using two-way ANOVA followed by Tukey's post hoc tests. Statistical significance was indicated as *p < 0.05, **p < 0.01, ***p < 0.001, ****p < 0.0001.


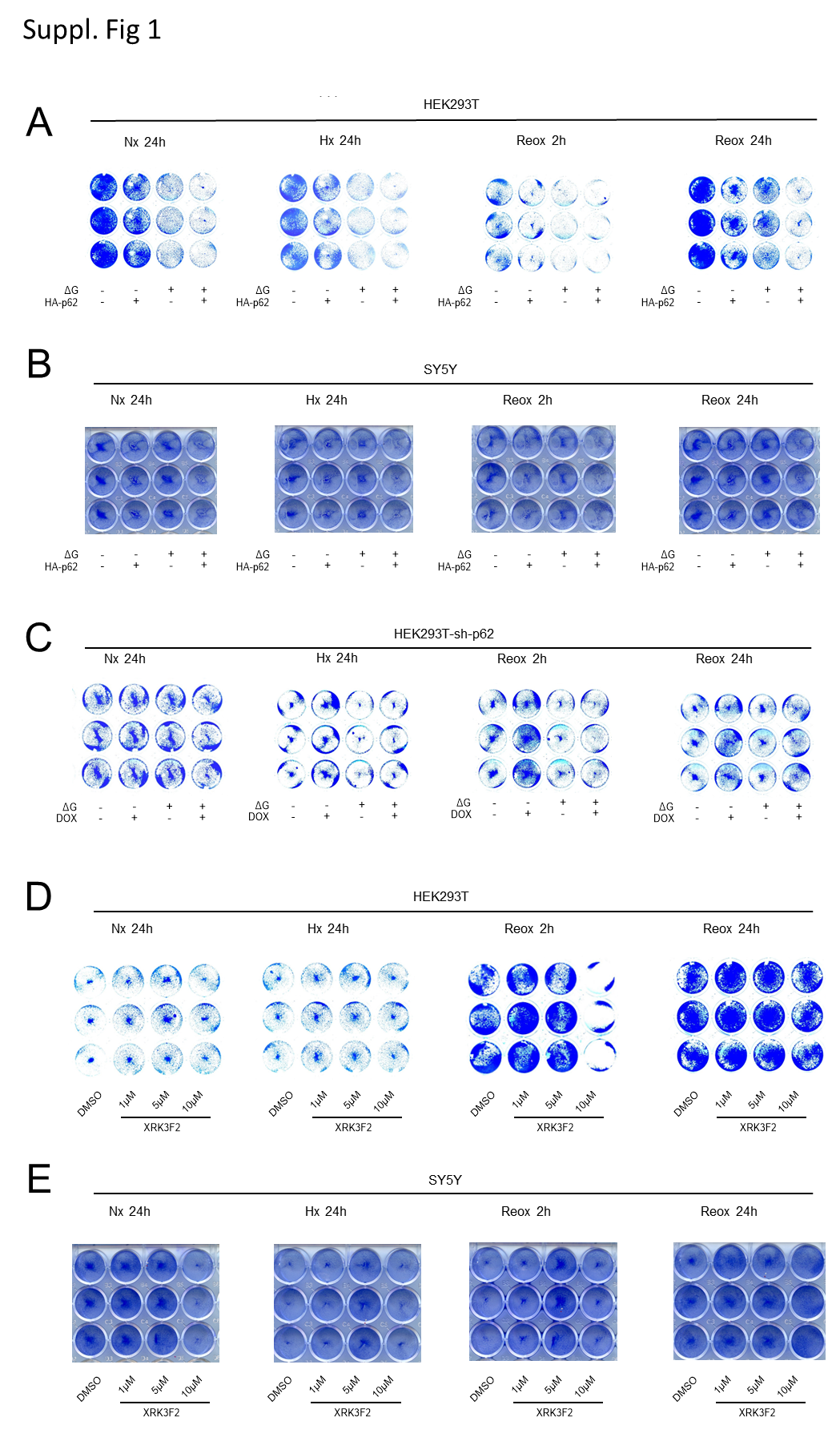


**Fig S2. Analysis of cell proliferation by Coomassie brilliant blue stainings.** HEK293 or SY5Y cells exposed to HA-p62 overexpression, sh-p62-mediated p62 knockdown or p62 deactivation by the pharmacological inhibitor XRK3F2 at different concentrations. All samples examined in this study under various oxygen and glucose deprivation conditions are shown.


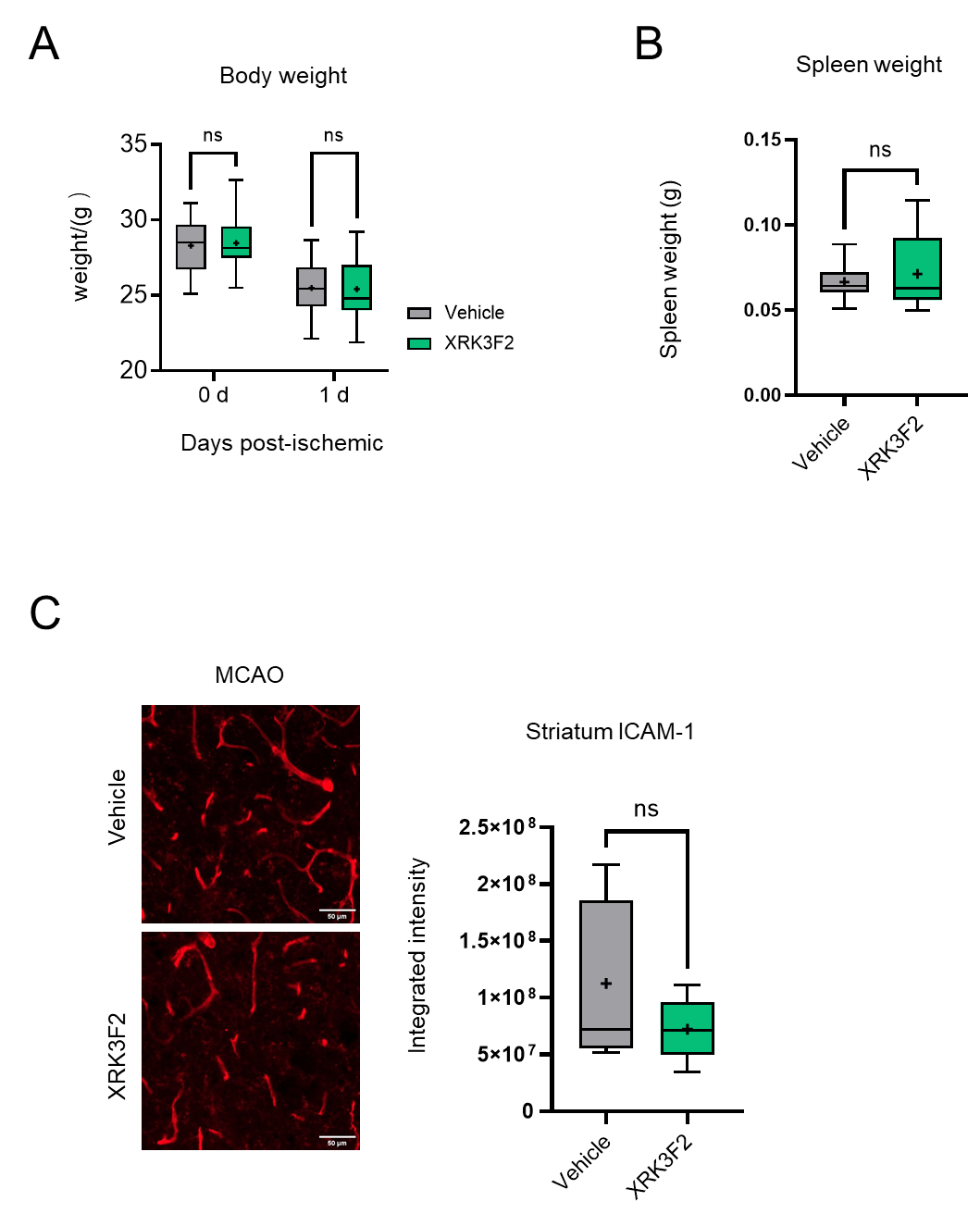


**Fig S3. Body weight, spleen weight and intercellular adhesion molecules-1 (ICAM1) abundance on ischemic microvessels were not influenced by pharmacological p62 deactivation in MCAO mice. (A)** Body weight and **(B)** spleen weight of mice exposed to MCAO that were sacrificed 24 hours after reperfusion (n=9 animals/ group). **(C)** Abundance of ICAM1 on brain microvessels analyzed by immunohistochemistry in the striatum of MCAO mice sacrificed 24 hours after reperfusion (n=9 animals/ group). Scale bar: 50 μm. Data are box plots indicating medians (lines inside boxes)/ means (crosses inside boxes) ± interquartile ranges, with minimum/maximum values shown as whiskers. Data were analyzed by Student's t-tests. No statistically significant differences were noted between groups.


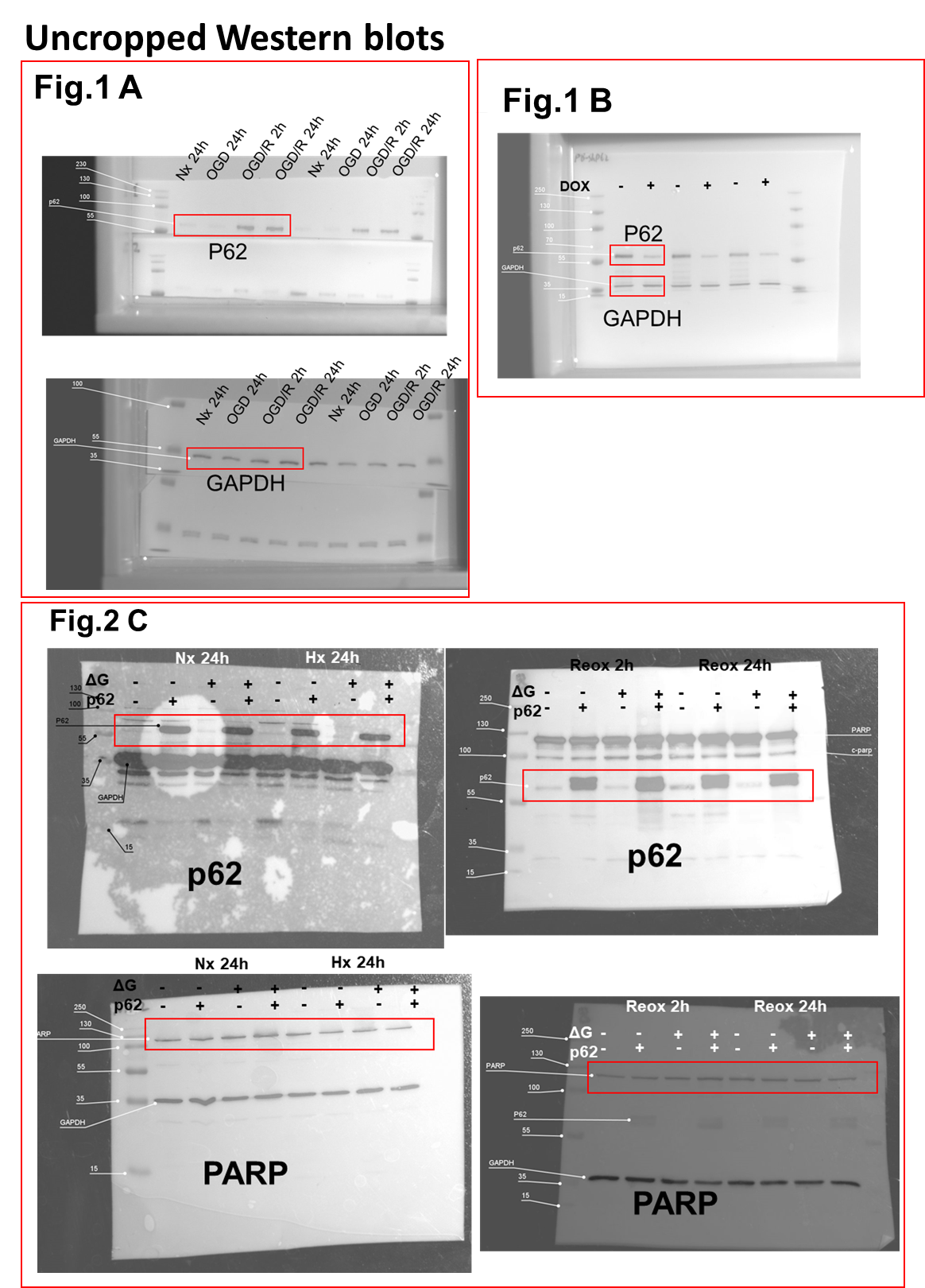


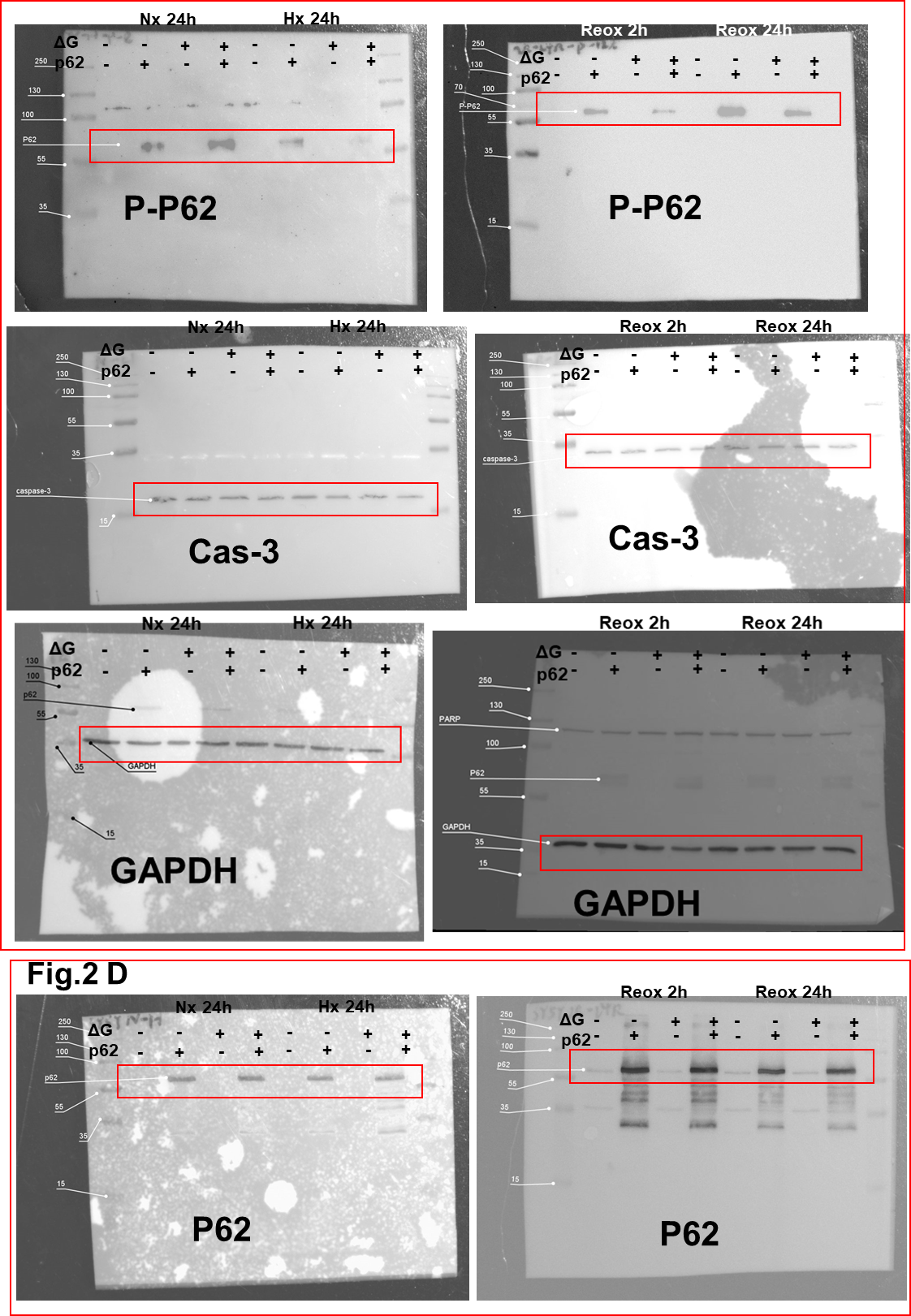


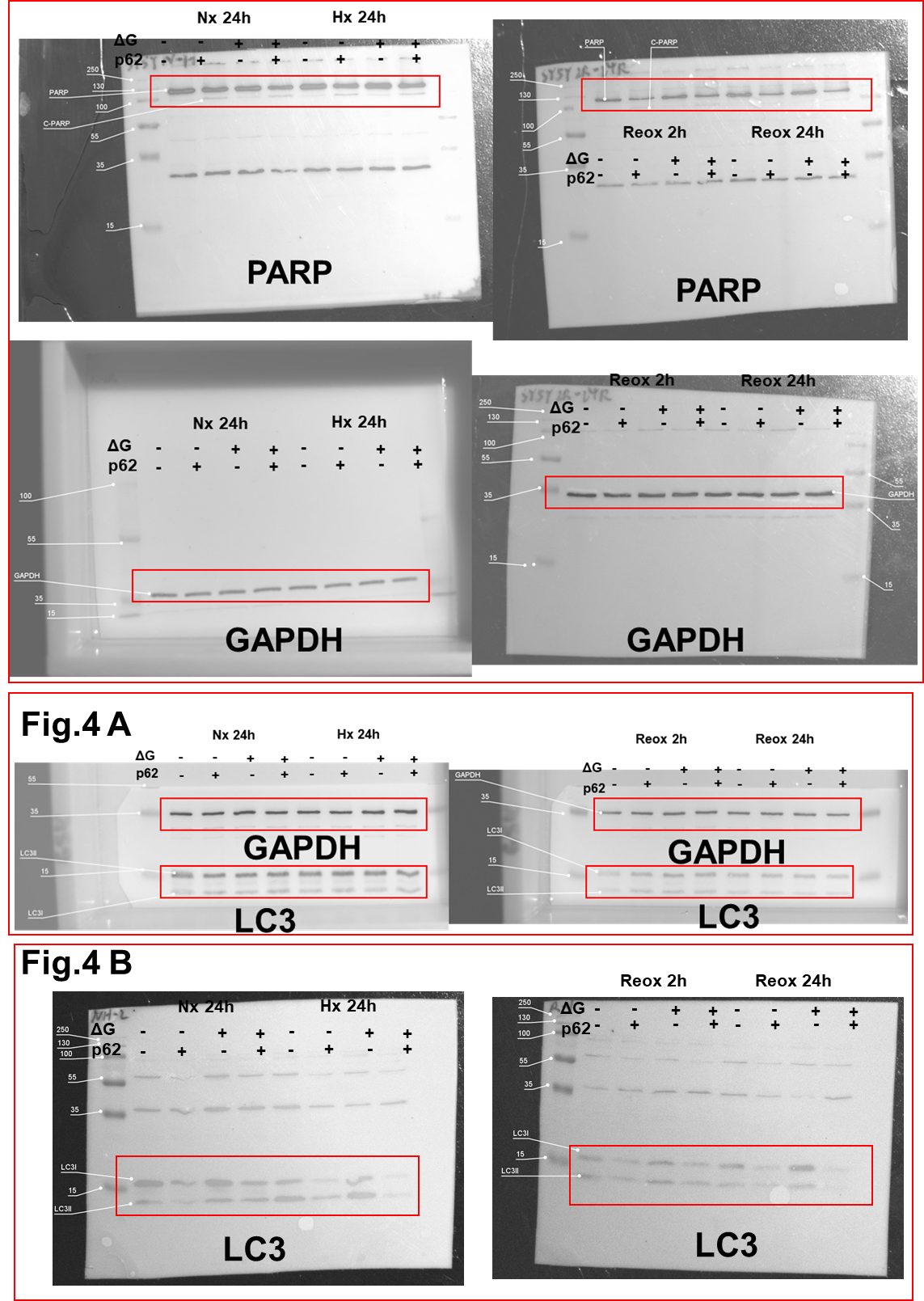


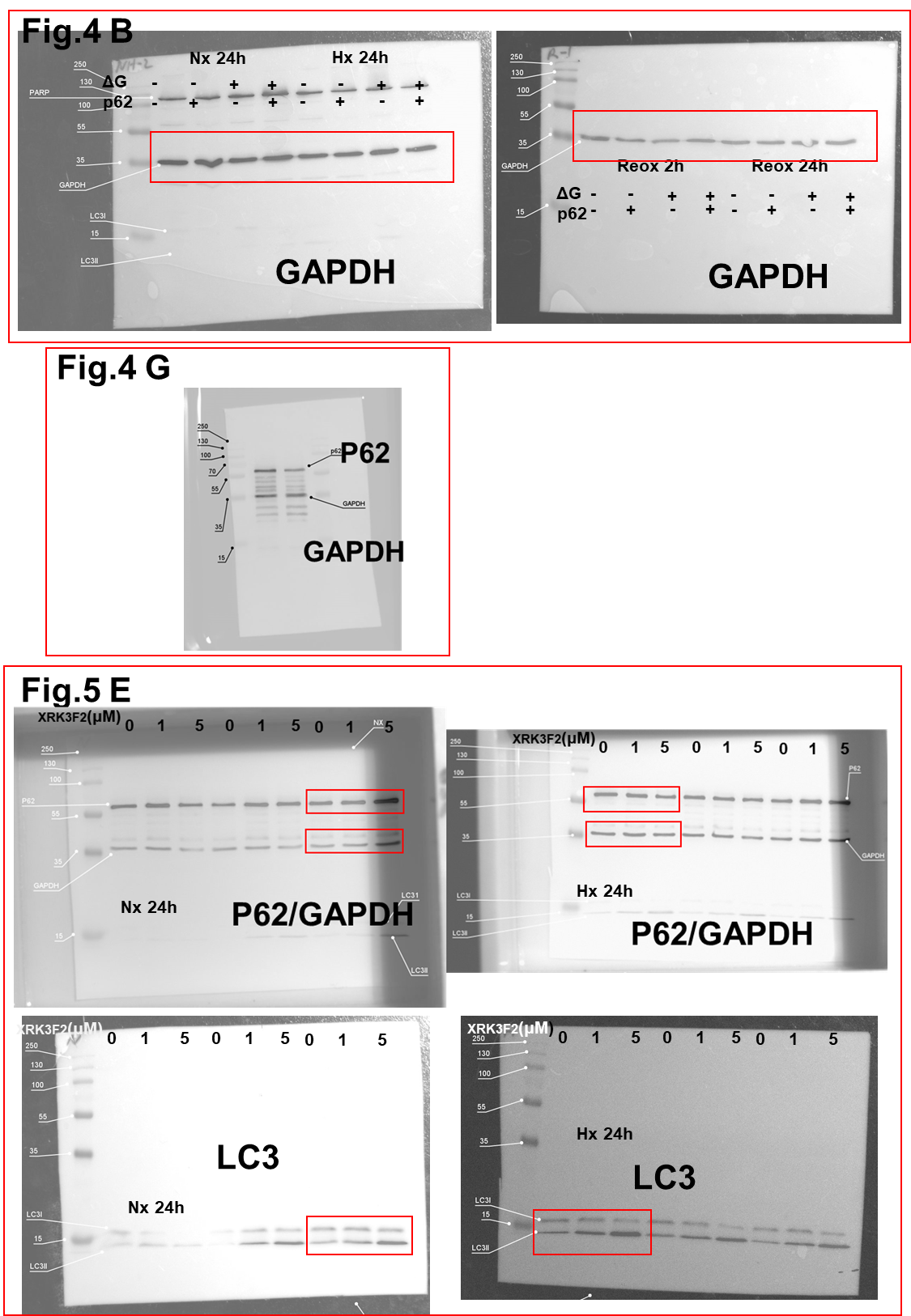


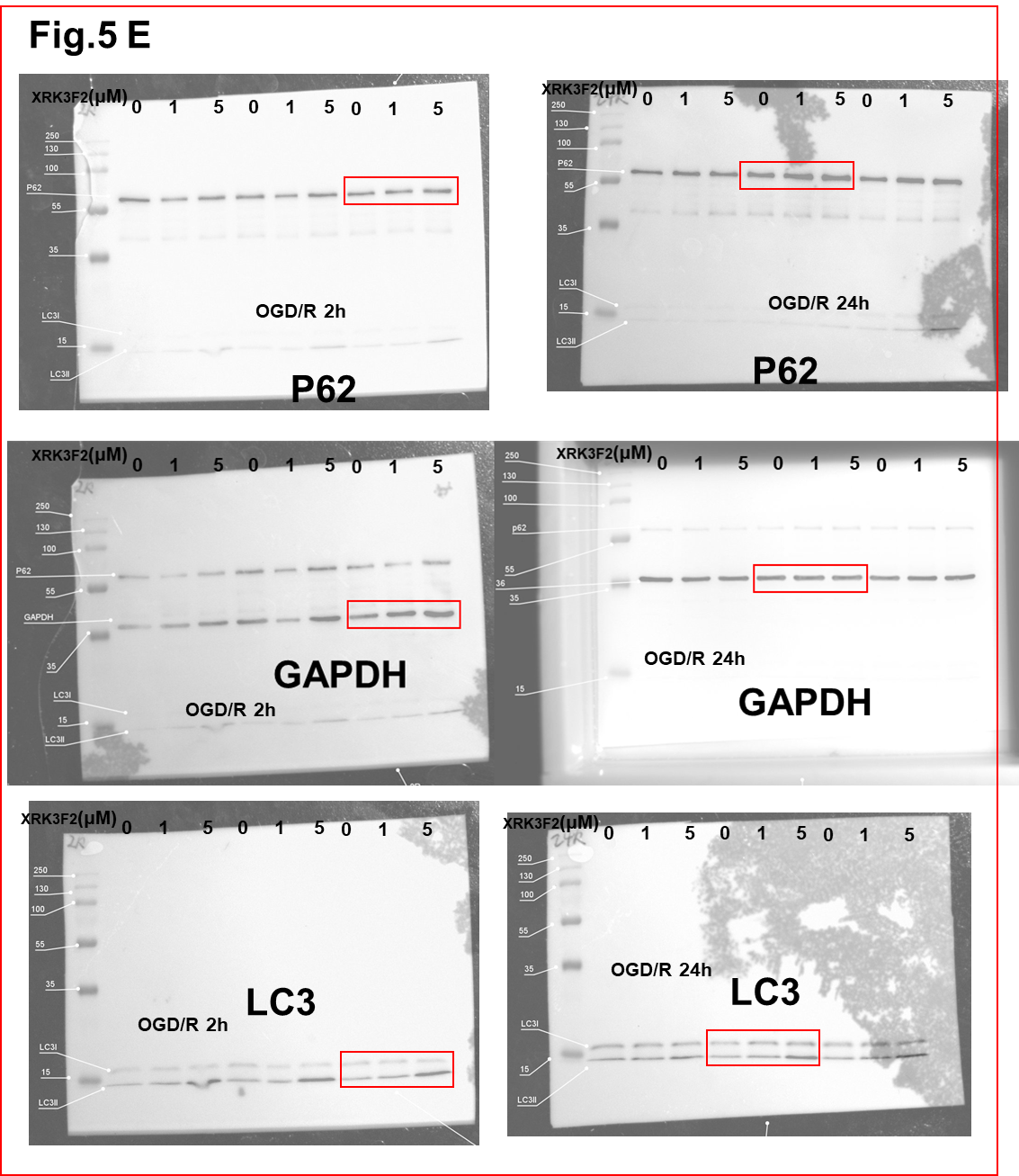


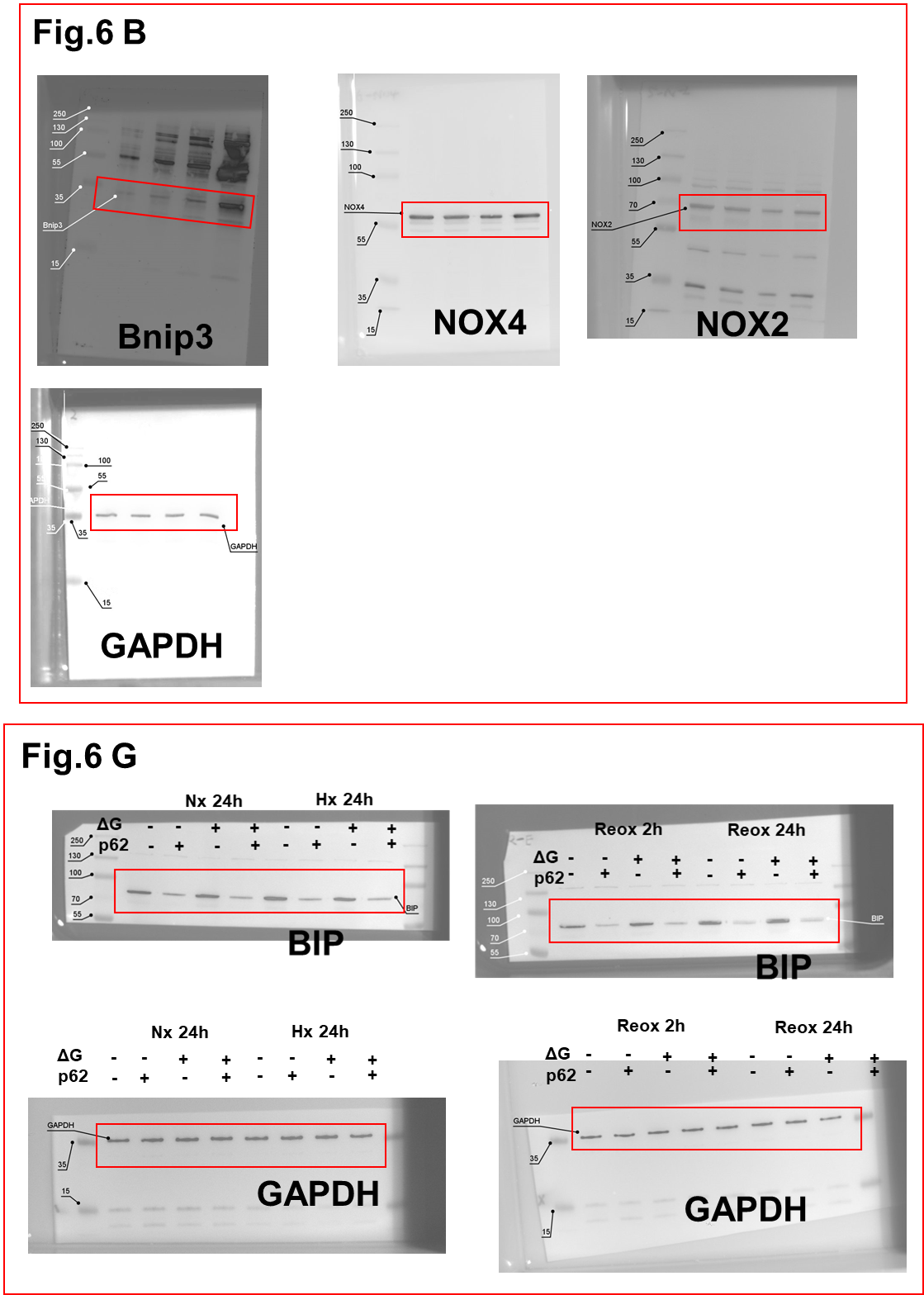


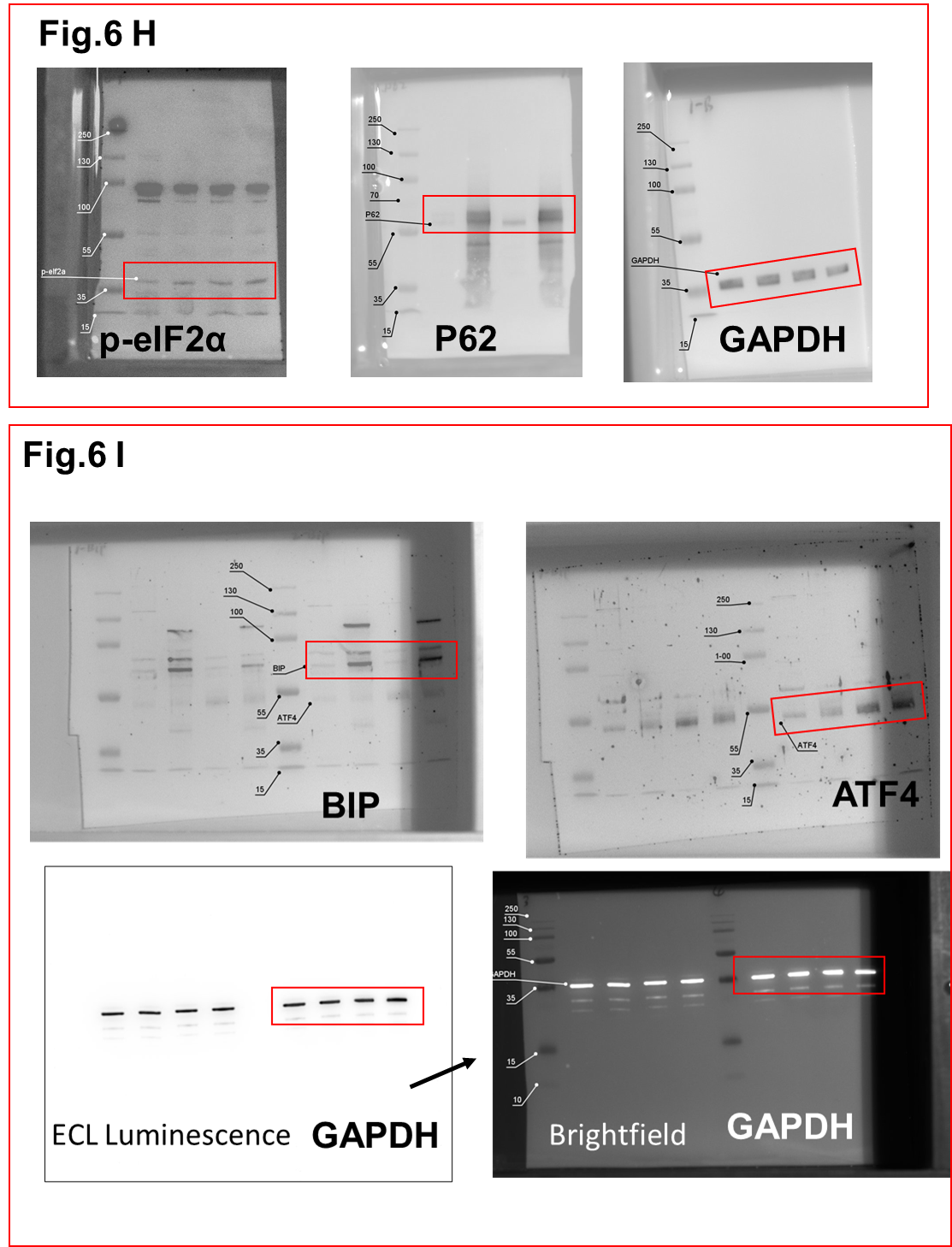


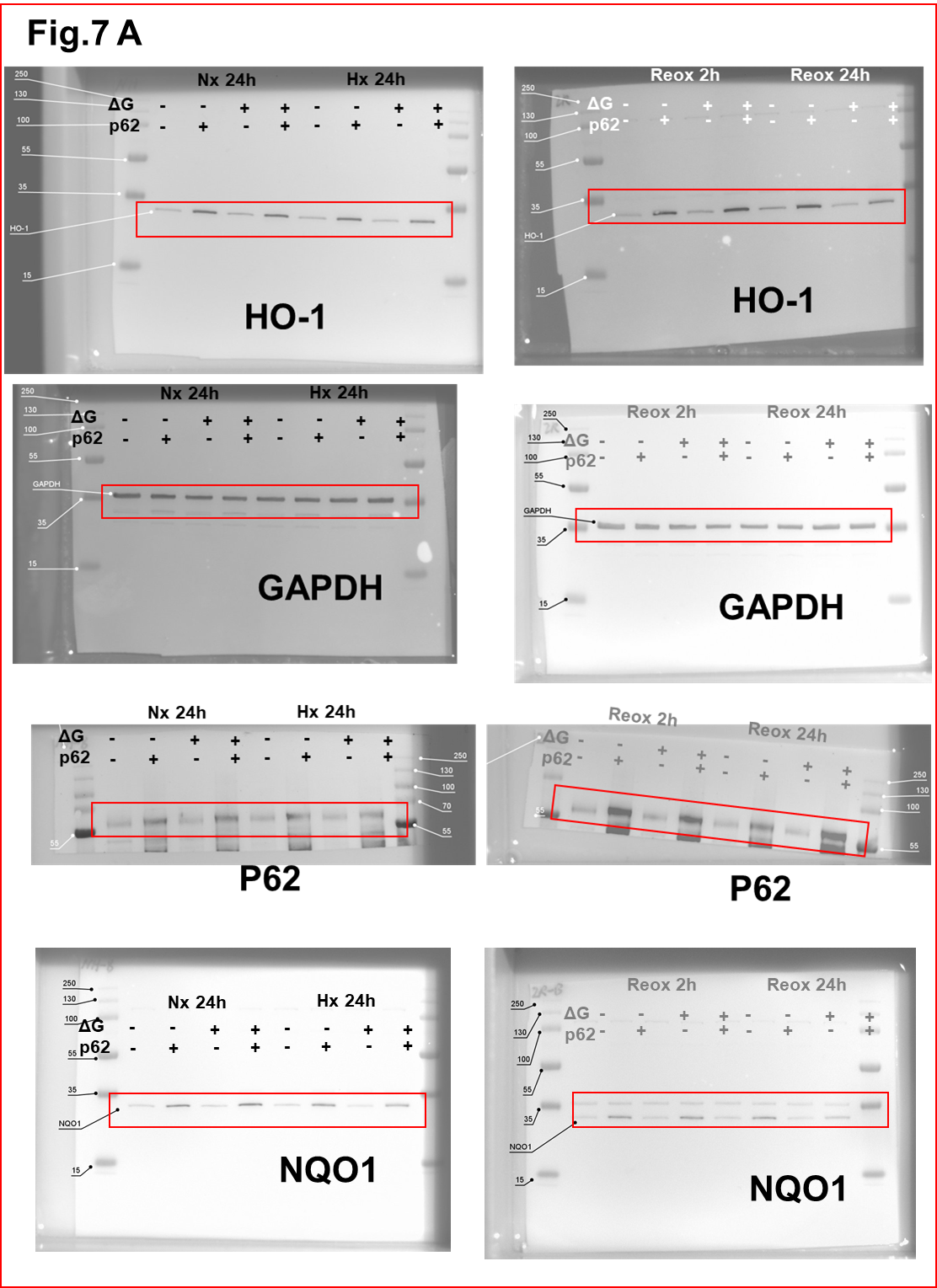


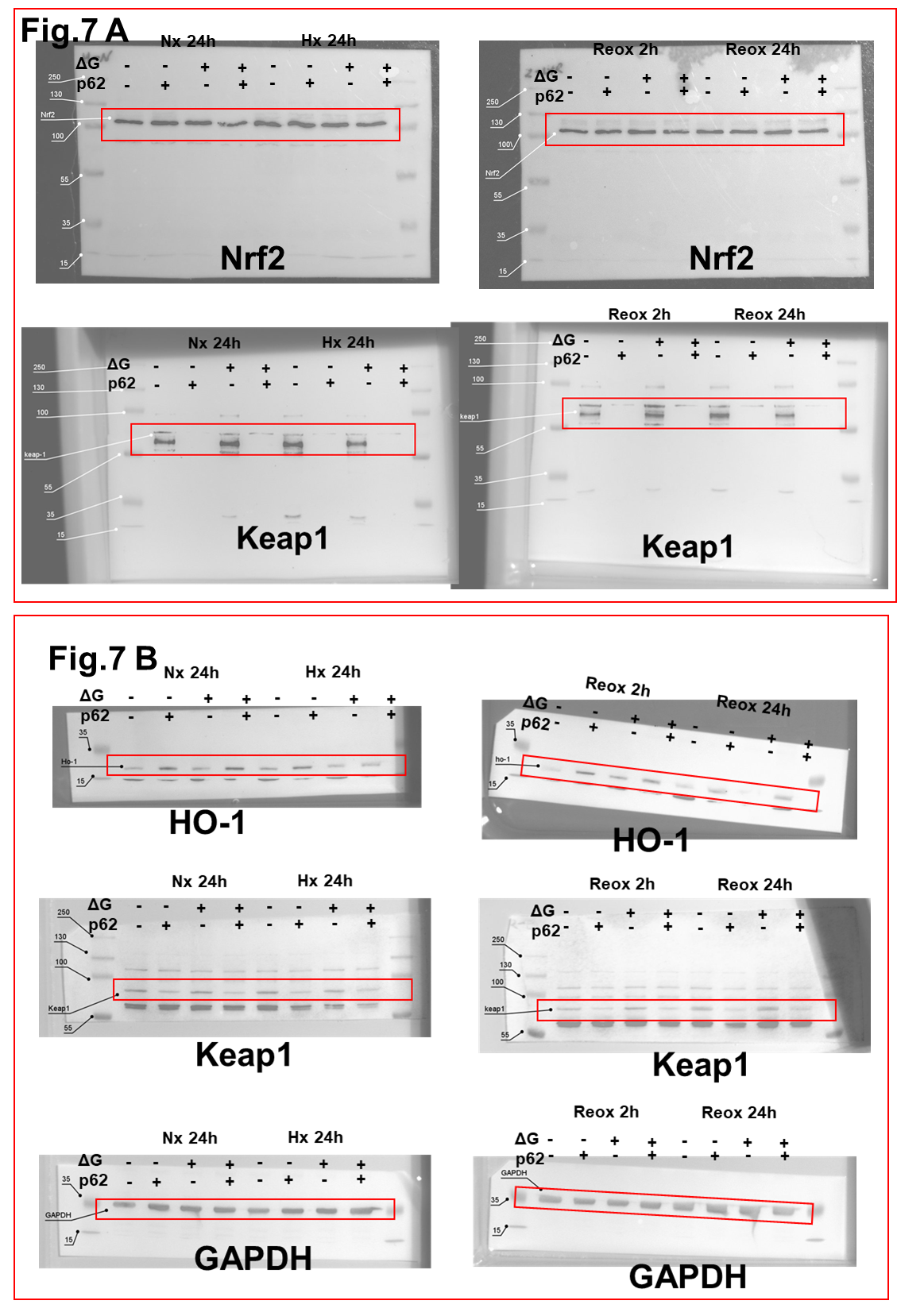


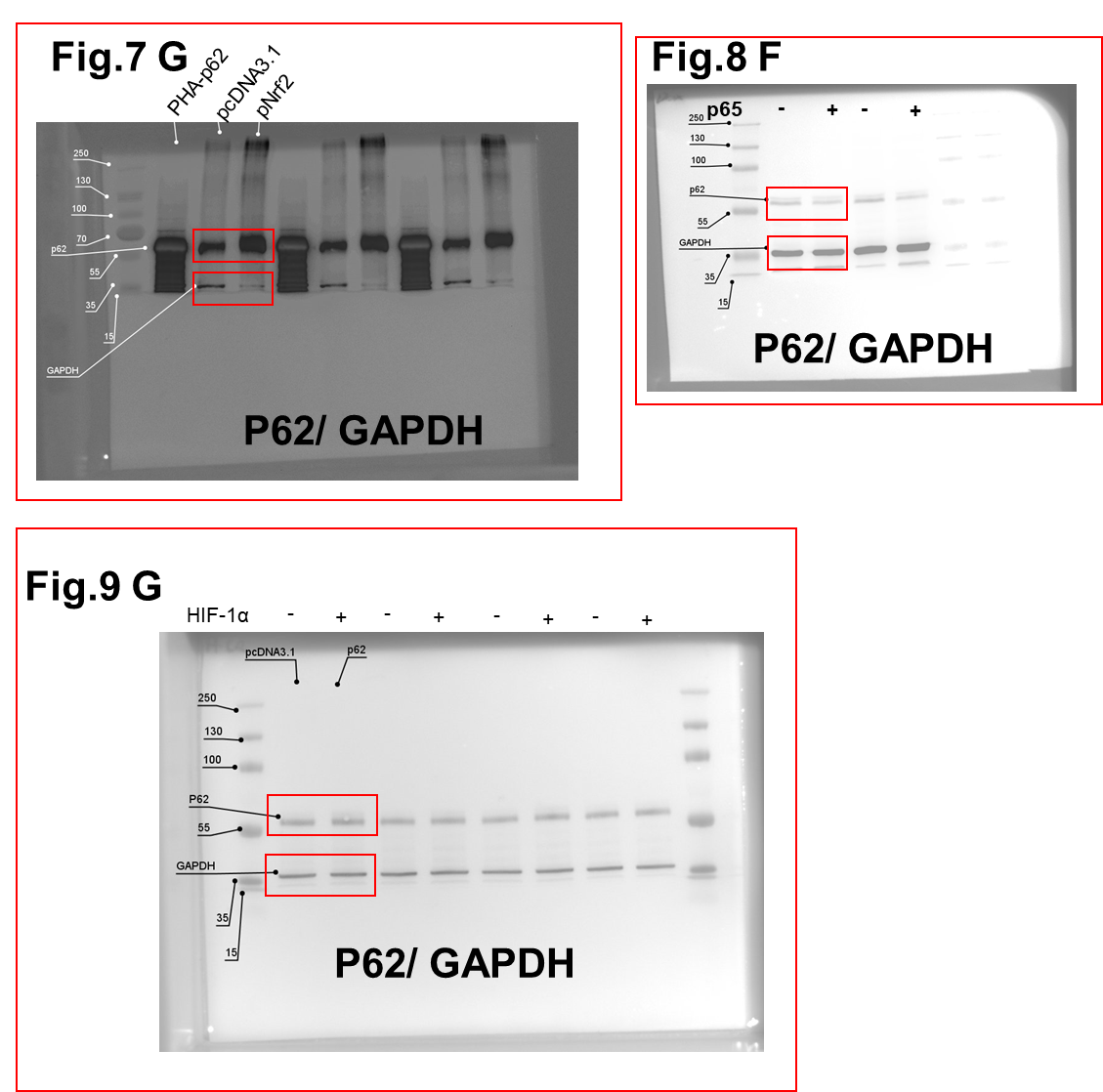


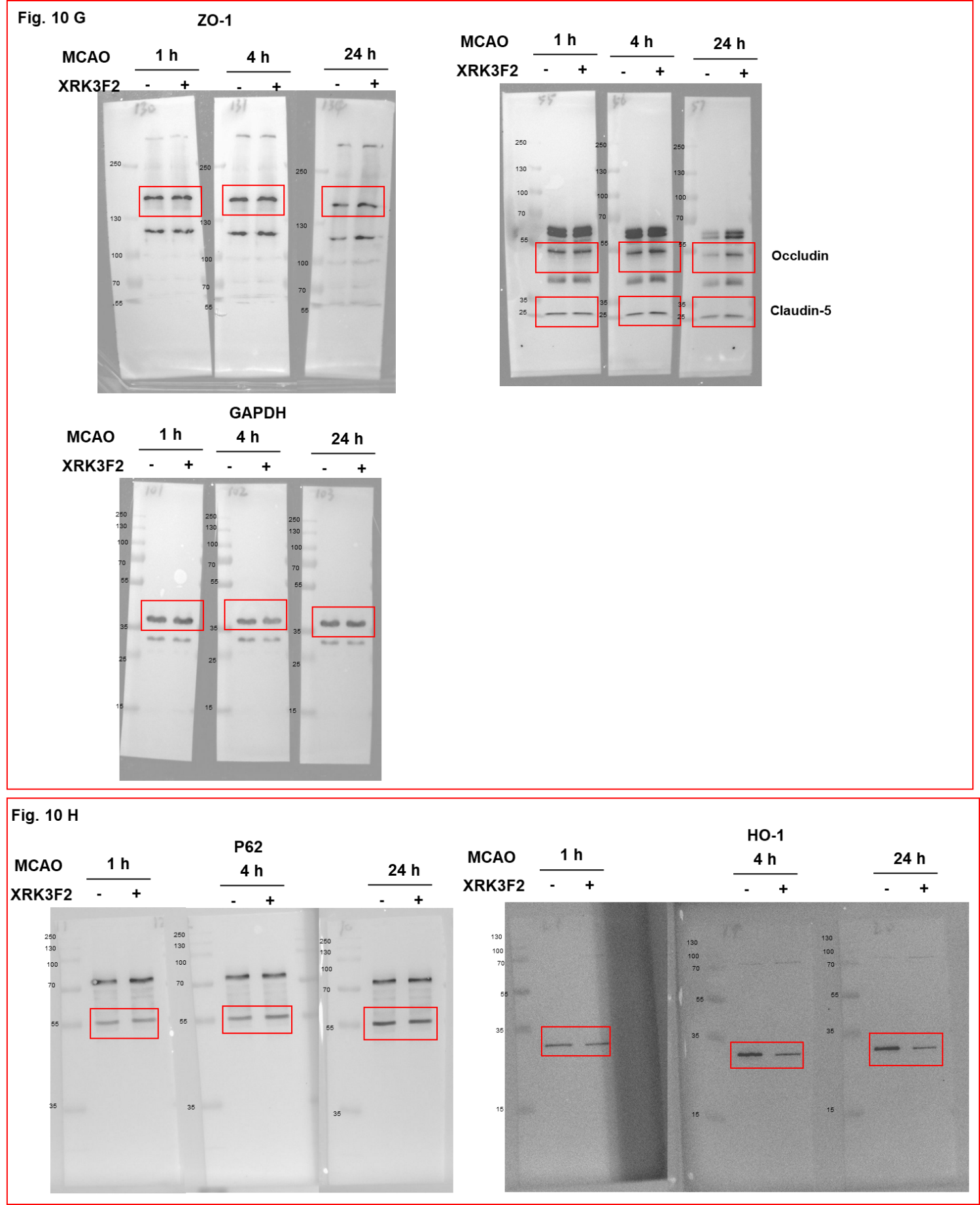


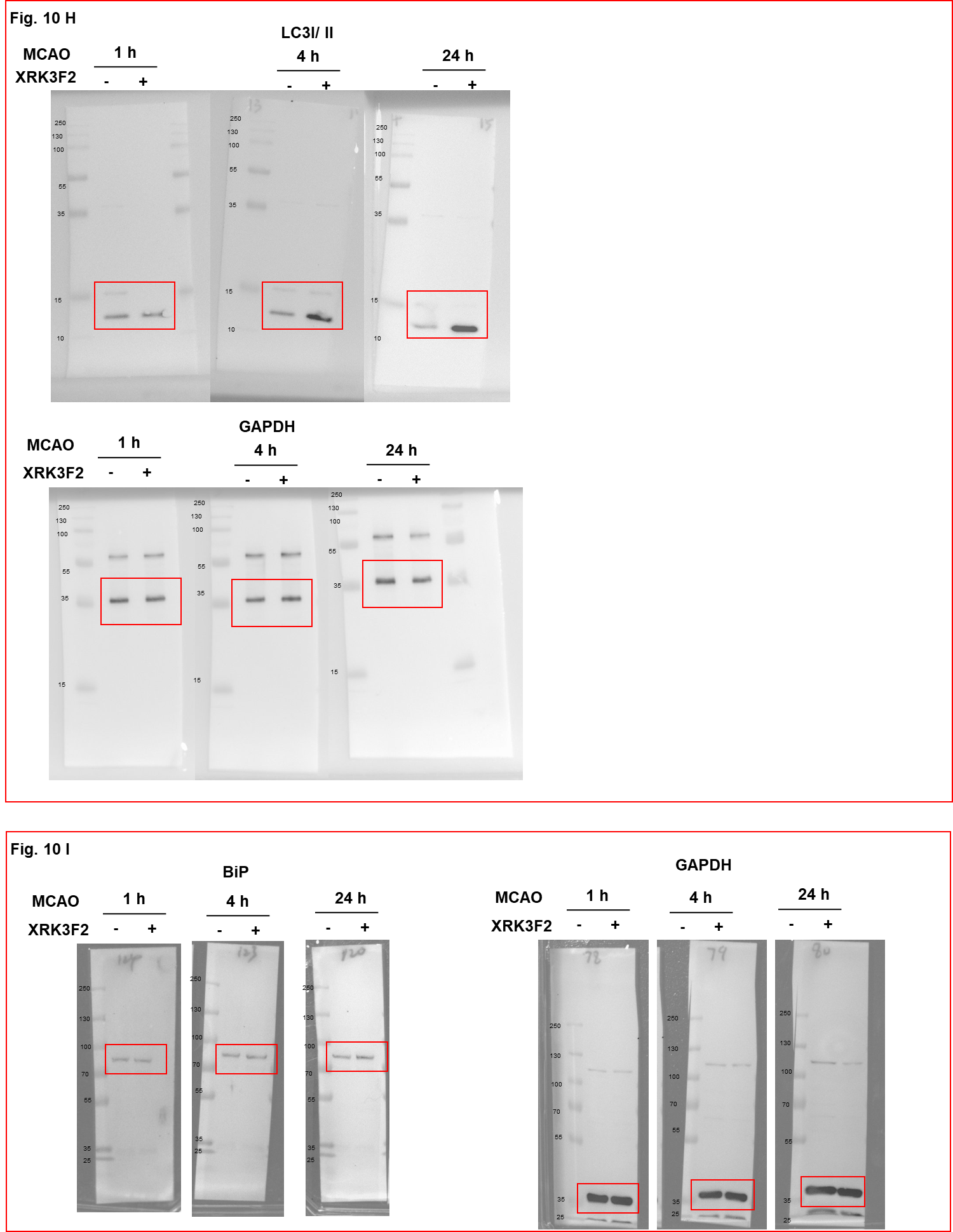

Supplement: Multimedia component 1 [file mmc1.docx]
